# Iron Nanoparticles on Porous Carbon Discs: Electrocatalysts with Over 30% Energy Efficiencies for the Production of Ammonia from Nitrate

**DOI:** 10.1002/advs.202514504

**Published:** 2025-09-29

**Authors:** Daon Park, Jinuk Choi, Ji Yong Shim, Yoon Kee Kim, Jina Park, Chang Wan Kang, So Young Park, Tae‐Yong An, Hyojung Lim, Subramani Surendran, Kyoung Chul Ko, Uk Sim, Seung Uk Son

**Affiliations:** ^1^ Department of Chemistry Sungkyunkwan University Suwon 16419 South Korea; ^2^ Hydrogen Energy Technology Laboratory Korea Institute of Energy Technology (KENTECH) Naju 58330 South Korea; ^3^ Department of Chemistry Education Chonnam National University Gwangju 61186 South Korea; ^4^ Research Institute NEEL Sciences, INC. Naju 58326 South Korea

**Keywords:** ammonia, carbon, electrocatalyst, iron, nitrate

## Abstract

Disc‐shaped precursor materials (Fe‐HBD) are prepared by the complexation of iron with 2,5‐dihydroxy‐1,4‐benzoquinone. Through the carbonization of Fe‐HBD at 700 °C, Fe nanoparticles on carbon disc matrixes (CD@Fe‐700) are synthesized. The CD@Fe‐700 with a high surface area of 424 m^2^ g^−1^ and micro/mesoporosity showed promising electrocatalytic performance in the nitrate reduction reaction (NO_3_RR) to ammonia with ammonia yield rates of 1.8–13.5 mg h^−1^ cm^−2^ at overpotentials of −0.2–−0.6 V (vs. RHE), reaching energy efficiencies up to 32.6–34.7%.

## Graphical abstract

Iron nanoparticles on carbon disc matrixes (CD@Fe) are engineered, showing a high surface area of 424 m^2^ g^−1^ and micro/mesoporosity. The CD@Fe showed promising electrocatalytic performance for the nitrate reduction to ammonia with ammonia yield rates of 1.8–13.5 mg h^−1^ cm^−2^ at overpotentials of −0.2–−0.6 V (vs. RHE), reaching energy efficiencies up to 32.6–34.7%.

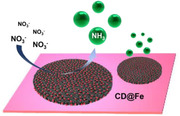

## 1 Introduction

Ammonia plays a vital role in sustaining modern society as one of the most important chemicals.^[^ ^1^, ^2^ ^]^ Thanks to the benefits of ammonia‐derived fertilizers, enhanced food production contributes to the growing human population.^[^ ^2^ ^]^ In addition, ammonia has been considered a candidate for carbon‐free fuels.^[^ ^3^ ^]^

**Figure 1:**
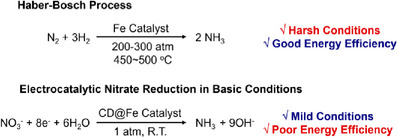
Haber‐Bosch Process and electrocatalytic nitrate reduction for the production of ammonia in basic conditions.

Ammonia is industrially produced by the Haber‐Bosch process, the reaction of nitrogen gas with hydrogen gas (**Figure** **1**).^[^ ^4^ ^]^ Although the Haber‐Bosch process requires a high temperature of 450–500 °C and a high pressure of 200–300 atm,^[^ ^4^ ^]^ the energy efficiency reached 35–60%. Iron was used as the catalyst for this reaction.^[^ ^4^ ^]^ It is worth noting that iron is the fourth most abundant element in the Earth's crust.^[^ ^5^ ^]^ Although ammonia production is of great importance, the Haber‐Bosch process has been recognized as a leading source of carbon dioxide emissions and one of the largest global energy consumers.^[^ ^6^ ^]^ As climate change, caused by greenhouse gases, continues to be a concern, there has been a growing need for greener processes of ammonia production.^[^ ^7^ ^]^

Recently, the electrocatalytic process for ammonia synthesis has gained considerable attention from scientists.^[^ ^8^ ^]^ Not only are the reaction conditions milder in electrocatalytic ammonia synthesis, but it also enables the use of electrical energy derived from sustainable sources. However, the electrocatalytic reduction of N_2_ to ammonia (NRR) suffers from low efficiency due to the poor interaction of N_2_ with electrocatalysts.^[^ ^9^ ^]^ The use of nitrate, instead of N_2_, is a promising method to obtain ammonia through electrocatalytic reduction (NO_3_RR) (Figure 1).^[^ ^10^ ^]^

Recently, Fe‐based electrocatalytic systems have been studied for NO_3_RR to ammonia.^[^ ^11^, ^12^, ^13^, ^14^, ^15^ ^]^ Nano‐engineered catalysts bearing Fe species have been synthesized, showing promising ammonia yield rates of 0.1–23.8 mg h^−1^ cm^−2^ (Table S1, Supporting Information).^[^ ^11^, ^12^, ^13^, ^14^, ^15^ ^]^ However, their energy efficiencies for the electrocatalytic NO_3_RR were significantly low in the range of 10∼30%, compared to those of the Haber‐Bosch process.^[^ ^4^, ^11^, ^12^, ^13^, ^14^, ^15^ ^]^ Thus, iron‐based electrocatalysts for the more energy‐efficient NO_3_RR require further exploration. In this work, we report the preparation of Fe‐carbon discs (CD@Fe) and their electrocatalytic performance in the production of ammonia from nitrate.

## 2 Results and Discussion

**Figure 2:**
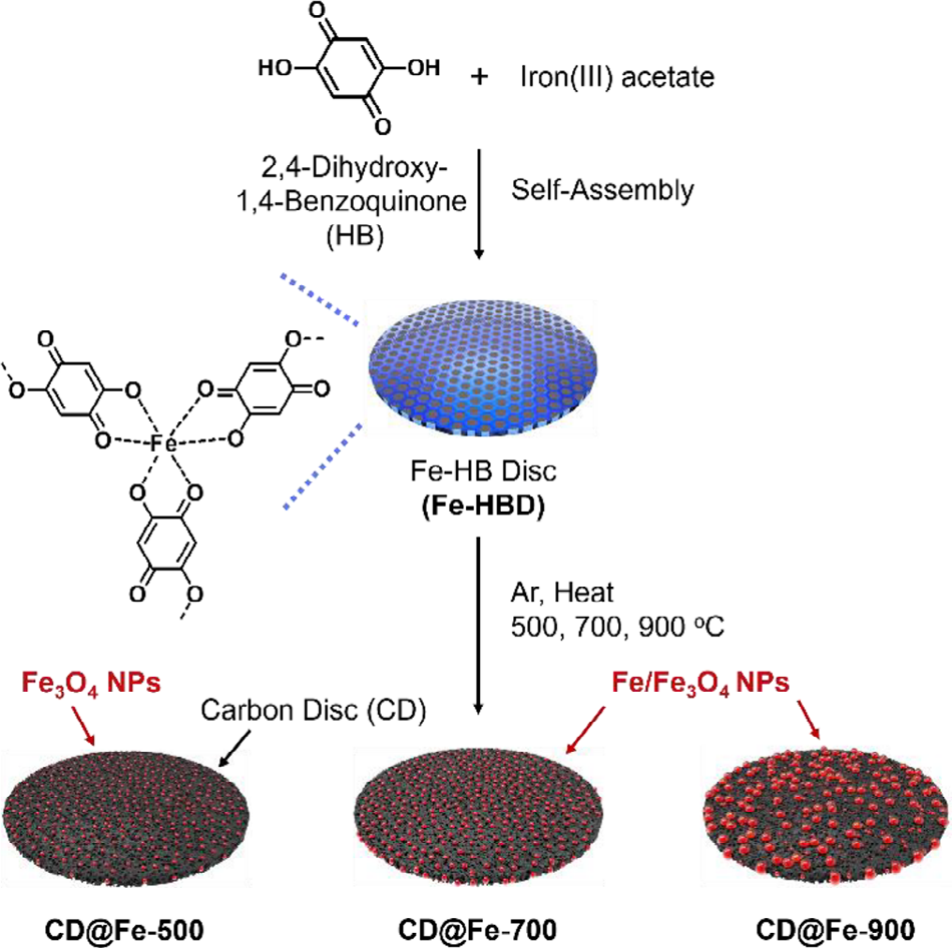
Synthesis of Fe‐HB disc precursor materials (Fe‐HBD) and carbon discs bearing iron‐based nanoparticles (CD@Fe).

**Figure** **2** illustrates synthetic schemes for CD@Fe electrocatalysts. First, the reaction of iron(III) acetate with 2,5‐dihydroxy‐1,4‐benzoquinone (HB) in dimethylformamide (DMF) at 80 °C resulted in the formation of precipitates, Fe‐HB precursor materials with a disc morphology (HBD) (Refer to the Supporting Information, SI for detailed synthetic procedures). According to thermogravimetric analysis (TGA), Fe‐HBD decomposes under argon at 175 °C (Figure S1, Supporting Information). The heat‐treatment of HBD at 500, 700, and 900 °C with the heating rate of 20 °C/min under argon resulted in the formation of CD@Fe materials, denoted as CD@Fe‐500, CD@Fe‐700, and CD@Fe‐900, respectively. Infrared (IR) absorption spectroscopy of Fe‐HBD showed the C─O vibration peaks at 1250 and 1359 cm^−1^ and aromatic C═C vibration peak at 1502 cm^−1^, indicating that Fe‐HBD contains HB building blocks (Figure S2, Supporting Information).^[^ ^16^ ^]^ While the IR spectrum of CD@Fe‐500 showed minor C─O vibration peaks, due to incomplete carbonization, those of CD@Fe‐700 and CD@Fe‐900 indicated complete carbonization (Figure S2, Supporting Information).

**Figure 3:**
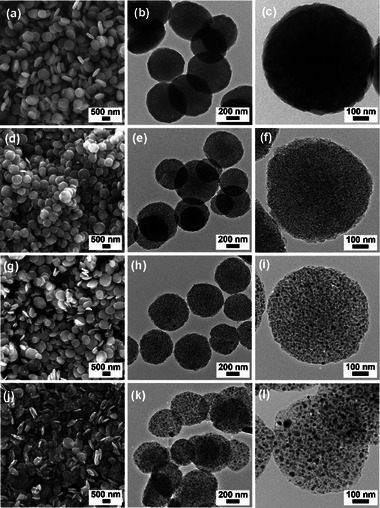
SEM images of a) Fe‐HBD, d) CD@Fe‐500, g) CD@Fe‐700, and j) CD@Fe‐900. TEM images of b,c) Fe‐HBD, e,f) CD@Fe‐500, h,i) CD@Fe‐700, and k,l) CD@Fe‐900.

The morphologies of Fe‐HBD and CD@Fe were investigated by scanning (SEM) and transmission electron microscopy (TEM) (**Figure** **3**). The SEM image of Fe‐HBD showed a disc shape with an average diameter of 720 nm and an average thickness of 240 nm (Figure 3a). In the TEM analysis of Fe‐HBD, the Fe‐based nanoparticles in the organic disc matrix were not observed, indicating that the Fe‐HBD is a molecular network (Figure 3b,c). Energy dispersive X‐ray spectroscopy (EDS)‐based elemental mapping studies on Fe‐HBD indicated the homogeneous distribution of carbon, oxygen, and iron across the disc materials, implying that Fe‐HBD was formed through the reaction of iron ions with HB building blocks (Figure S3, Supporting Information).

The SEM analysis showed that while CD@Fe‐500 maintained the original disc shape of Fe‐HBD, an average diameter and an average thickness were significantly reduced to 705 and 130 nm, respectively, through the carbonization of organic components in precursor materials (Figure 3d). Interestingly, TEM analysis of CD@Fe‐500 showed the formation of small iron‐based nanoparticles with sizes of 5–7 nm in a carbon disc matrix (Figure 3e,f). The small iron‐based nanoparticles were homogeneously distributed over the carbon discs (Figure 3f). In the case of CD@Fe‐700, while the disc shape was maintained, an average diameter and an average thickness were further reduced to 640 and 110 nm, respectively (Figure 3g). While the iron‐based nanoparticles maintained the homogeneous distribution over carbon disc matrixes, the sizes of iron nanoparticles increased to 10–15 nm (Figure 3h,i). As expected, the SEM image of CD@Fe‐900 showed that the average diameter and thickness were further reduced to 608 and 90 nm, respectively (Figure 3j). The size distribution of the iron‐based nanoparticles in CD@Fe‐900 became broad in the range of 10–60 nm, indicating that the Ostwald ripening occurred during the growth of iron‐based nanoparticles (Figure 3k,l).^[^ ^17^ ^]^ TEM images of carbon discs obtained through iron‐etching from CD@Fe confirmed the existence of carbon matrixes (Figure S4, Supporting Information).

**Figure 4:**
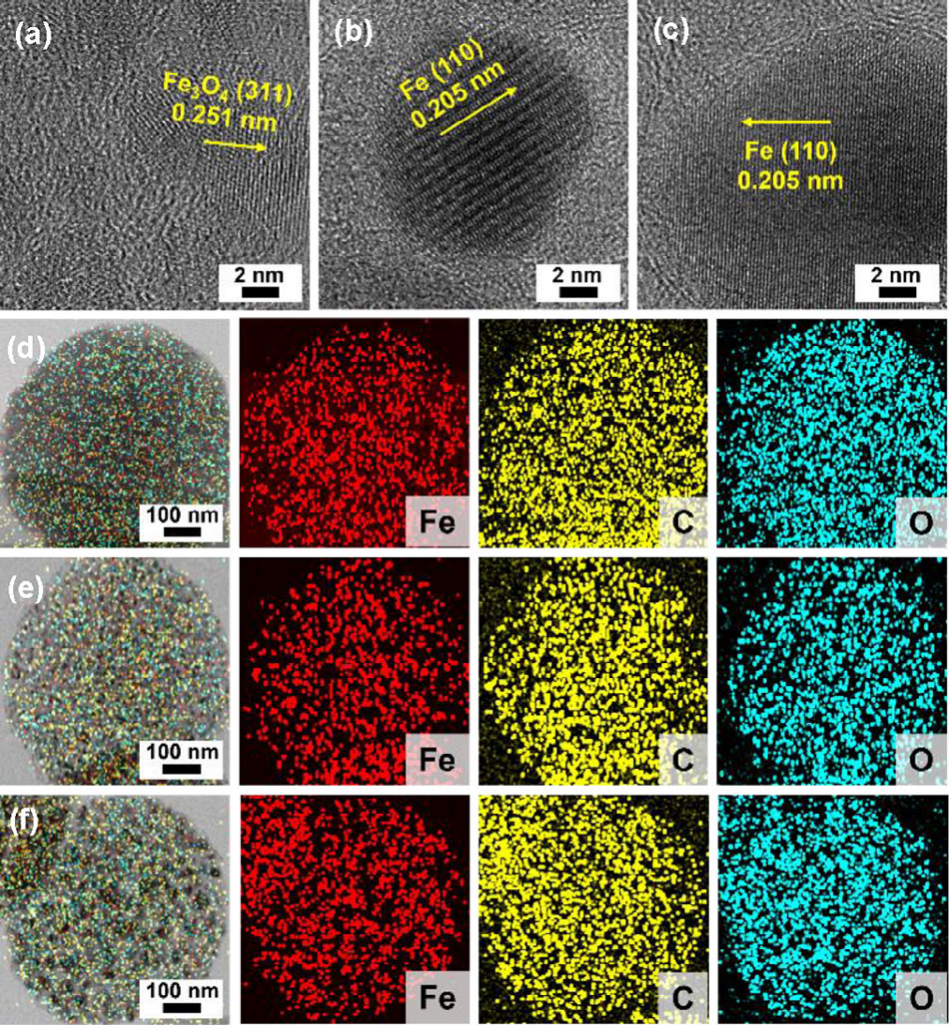
HR‐TEM images of a) CD@Fe‐500, b) CD@Fe‐700, and c) CD@Fe‐900. EDS‐based elemental mapping images of d) CD@Fe‐500, e) CD@Fe‐700, and f) CD@Fe‐900.

High‐resolution TEM analysis (HR‐TEM) was conducted to characterize the chemical compositions of iron‐based nanoparticles on CD@Fe materials (**Figure** **4**a–c). In the case of CD@Fe‐500, while carbon matrixes are amorphous, the interplane distances of 0.251 nm were exclusively observed on the iron‐based nanoparticles, corresponding to the (311) crystalline plane of Fe_3_O_4_ (Figure 4a).^[^ ^18^ ^]^ We speculate that the formed metallic Fe nanoparticles were oxidized through the reaction with air to form Fe_3_O_4_ nanoparticles. It is noteworthy that freshly prepared CD@Fe‐500 often ignites when treated under air. To prevent ignition, the freshly prepared CD@Fe‐500 must be gently exposed to air to induce slow oxidation.

The HR‐TEM analysis on iron‐based nanoparticles of CD@Fe‐700 showed exclusively the interplane distance of 0.205 nm, corresponding to the (110) crystalline plane of α‐metallic Fe (Figure 4b).^[^ ^19^ ^]^ This indicates that the Fe(III) ions were reduced to zerovalent iron during the carbonization of Fe‐HBD. We speculate that the generated carbon acted as a reductant, as the industrial iron is engineered using carbon as a reductant to produce carbon dioxide. Similar to CD@Fe‐700, the (110) crystalline plane of α‐metallic Fe with the interplane distance of 0.205 nm was exclusively observed in the HR‐TEM analysis of iron‐based nanoparticles of CD@Fe‐900 (Figure 4c). While the significant iron oxides appear to exist on the surface of iron‐based nanoparticles in the HR‐TEM images of CD@Fe‐700 and CD@Fe‐900, their crystalline structures could not be confirmed due to the poor crystallinity.

Chemical compositions and their distribution in CD@Fe were further investigated by EDS‐based elemental mapping studies (Figure 4d–f). As shown in Figure 4d, iron, carbon, and oxygen were homogeneously distributed over CD@Fe‐500 discs. In comparison, while carbons were distributed over CD@Fe‐700 and CD@Fe‐900 discs, the iron and oxygen were present on the Fe‐based nanoparticles, confirming that the chemical structures of CD@Fe consist of iron/iron oxide nanoparticles and the carbon matrixes (Figure 4e,f).

**Figure 5:**
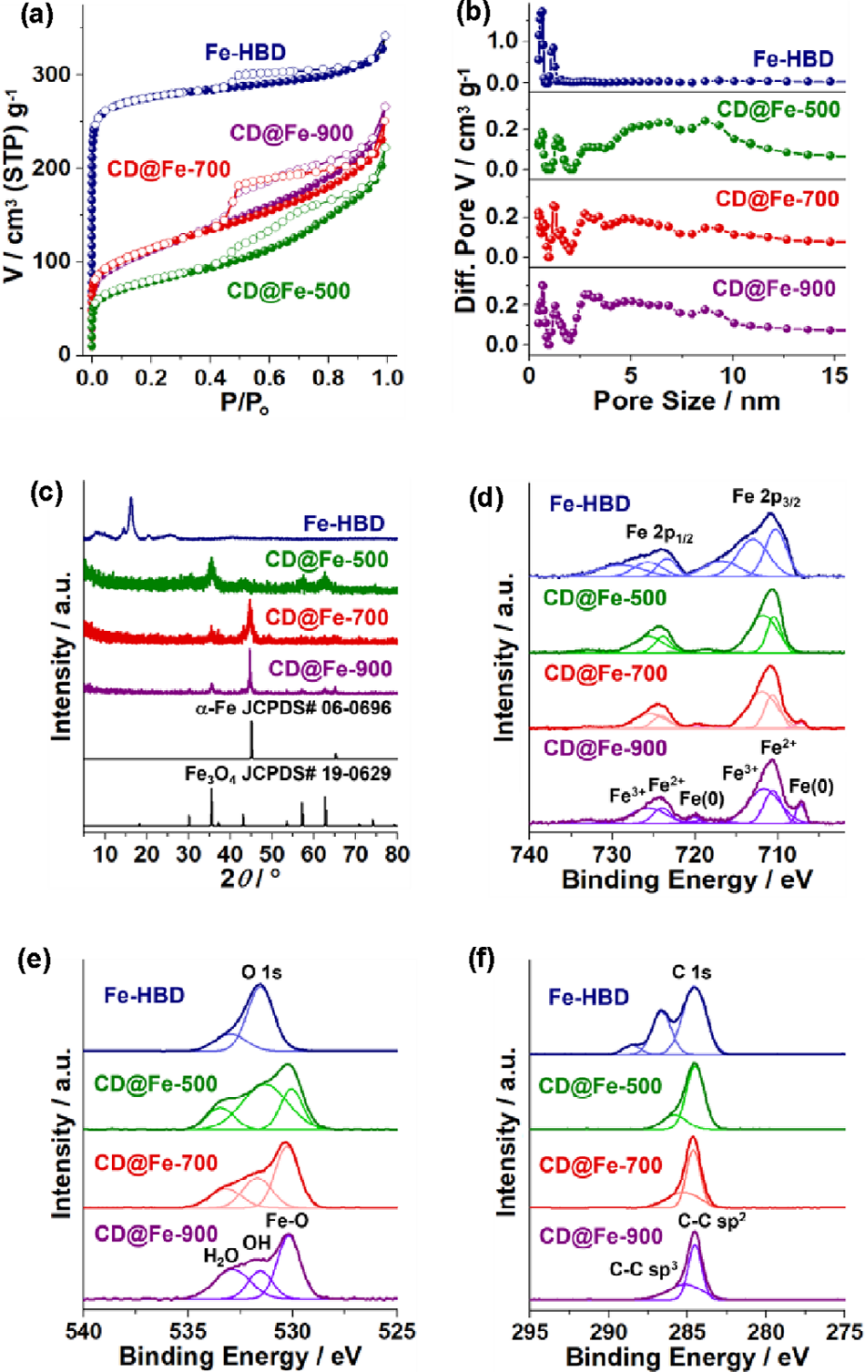
a) N_2_ adsorption‐desorption isotherm curves obtained at 77 K, b) pore size distribution diagrams based on NLDFT method, (c) PXRD patterns, d–f) XPS Fe 2p, O 1s, and C 1s orbitals spectra of Fe‐HBD, CD@Fe‐500, CD@Fe‐700, and CD@Fe‐900.

The porosity and surface areas of Fe‐HBD and CD@Fe materials were investigated by the analysis of N_2_ adsorption‐desorption isotherm curves based on Brunauer‐Emmett‐Teller (BET) theory (**Figure** **5**a; Table S2, Supporting Information). The Fe‐HBD showed a high surface area of 1095 m^2^ g^−1^ with a total pore volume (V_t_) of 0.41 cm^3^ g^−1^. The microporous feature of Fe‐HBD was analyzed by the non‐local density functional theory (NLDFT) method, showing that the main pore sizes are less than 2 nm with a micropore volume (V_mic_) of 0.36 cm^3^ g^−1^ (Figure 5b).

In comparison, all CD@Fe materials showed significant mesoporosity with pore size distributions in the range of 2–15 nm, in addition to a microporous feature (Figure 5a,b). The surface area of CD@Fe‐500 was analyzed to be 282 m^2^ g^−1^ (Table S2, Supporting Information). While the V_t_ of CD@Fe‐500 was 0.22 cm^3^ g^−1^, it was analyzed as the combination of V_mic_ and mesopore volume (V_meso_) of 0.040 and 0.18 cm^3^ g^−1^, respectively. The surface areas of CD@Fe‐700 and CD@Fe‐900 increased to 424 and 400 m^2^ g^−1^, respectively (Table S2, Supporting Information). While the V_t_ of CD@Fe‐700 was 0.28 cm^3^ g^−1^, it was analyzed as the combination of the V_mic_ and V_meso_ of 0.048 and 0.23 cm^3^ g^−1^, respectively. Similarly, while the V_t_ of CD@Fe‐900 was 0.27 cm^3^ g^−1^, it was analyzed as the combination of V_mic_ and V_meso_ of 0.039 and 0.23 cm^3^ g^−1^, respectively.

Although Fe‐HBD exhibits crystallinity, we were unable to obtain its single crystals (Figure 5c). However, the powder X‐ray diffraction (PXRD) pattern of Fe‐HBD matched that (CCDC# 2224779) of the known 2:3 complex of Fe ions and HB building blocks. Combustion‐based elemental analysis and inductively coupled plasma‐atomic emission spectroscopy (ICP‐AES) revealed that the carbon, oxygen, hydrogen, and iron contents of Fe‐HBD were 38.6, 38.3, 2.6, and 20.3 wt.%, respectively, which are close to the theoretical values (C: 38.5, O: 39.9, H: 1.8, Fe: 19.9 wt.%) for the expected 2:3 complex ([C_18_H_6_O_12_Fe⋅2H_2_O]_∞_) of iron ions and HB building blocks (Refer to the suggested network structure of Fe‐HBD in Figure 2).^[^ ^20^ ^]^

The PXRD pattern of CD@Fe‐500 showed the diffraction peaks at 30.2, 35.5, 43.2, 53.4, 57.1, and 62.7 ° (2*θ*), corresponding to the (220), (311), (400), (422), (511), and (440) crystal planes of Fe_3_O_4_ (JCPDS# 19‐0629).^[^ ^21^ ^]^ In comparison, the PXRD patterns of CD@Fe‐700 and CD@Fe‐900 showed the mixture of diffraction peaks of metallic α‐Fe (JCPDS# 06‐0696) and Fe_3_O_4_. The main diffraction peaks of CD@Fe‐700 and CD@Fe‐900 were observed at 44.6 and 65.0 ° (2*θ*), corresponding to the (110) and (200) crystal planes of metallic α‐Fe.^[^ ^22^ ^]^

Through X‐ray photoelectron studies (XPS), the chemical surroundings of iron, oxygen, and carbon in Fe‐HBD and CD@Fe materials were analyzed (Figure 5d–f). The XPS Fe 2p orbitals spectrum of Fe‐HBD showed three kinds of Fe 2p_3/2_ orbital peaks at 710.3, 713.0, and 716.8 eV, in addition to Fe 2p_1/2_ orbital peaks at 723.6, 726.6, and 730.9 eV, respectively, corresponding to O‐Fe in octahedral complexes, O‐Fe in unsaturated complexes, and surface Fe ionic species (Figure 5d; Figure S5. Supporting Information).^[^ ^23^ ^]^ In the XPS O 1s orbital spectrum of Fe‐HBD, two kinds of peaks were observed at 531.5 and 533.0 eV, corresponding to phenoxy oxygen and carbonyl oxygen species, respectively (Figure 5e).^[^ ^23^ ^]^ In addition, three kinds of XPS C 1s orbital peaks were observed at 284.6, 286.7, and 288.5 eV, corresponding to aromatic carbons of C‐C sp^2^, C─O, and C═O species, respectively (Figure 5f).^[^ ^23^ ^]^

In the XPS spectra of CD@Fe‐500, CD@Fe‐700, and CD@Fe‐900, while Fe 2p_3/2_ and 2p_1/2_ orbital peaks of Fe^2+^ species appeared at 710.5–710.7 and 723.8–724.1 eV, those of Fe^3+^ species were observed at 711.6–711.9 and 725.4–725.7 eV, respectively (Figure 5d).^[^ ^24^ ^]^ While the Fe 2p_3/2_ and Fe 2p_1/2_ orbital peaks of zerovalent Fe were not detected in the XPS spectrum of CD@Fe‐500, those of CD@Fe‐700 and CD@Fe‐900 were observed at 707.2–707.3 and 720.0–720.1 eV, respectively.^[^ ^21^ ^]^ The XPS O 1s orbital spectra of CD@Fe‐500, CD@Fe‐700, and CD@Fe‐900 showed three kinds of peaks at 530.1–530.3, 531.3–531.7, and 532.9–533.4 eV, corresponding to Fe─O, Fe─OH on surface, and adsorbed water, respectively (Figure 5e).^[^ ^24^, ^25^ ^]^ Interestingly, the contents of Fe─OH gradually decreased from 58.0% (CD@Fe‐500) to 29.5 (CD@Fe‐700) and 21.1% (CD@Fe‐900), respectively, due to the enhanced dehydration to form iron oxides. Two kinds of C 1s orbital peaks of CD@Fe were observed at 284.5–284.6 and 285.1–285.8 eV, corresponding to sp^2^ and sp^3^ carbon species, respectively (Figure 5f).^[^ ^24a^ ^]^

According to XPS analysis, CD@Fe‐500 contained only cationic Fe species, whereas the molar ratio of zerovalent Fe to cationic Fe species of Fe_3_O_4_ in CD@Fe‐700 and CD@Fe‐900 gradually increased from 3.9:96.1 to 8.8:91.2 (Figure S5, Supporting Information). However, it can be noted that the XPS analysis reflects the chemical surroundings near the surface. Through the combustion‐based elemental analysis and ICP‐AES studies, the contents of carbon, oxygen, hydrogen, and iron in CD@Fe were analyzed. While the carbon contents gradually increased from 42.0 wt.% (CD@Fe‐500) to 45.6 (CD@Fe‐700) and 47.2 wt.% (CD@Fe‐900), the oxygen contents gradually decreased from 21.6 wt.% (CD@Fe‐500) to 9.9 (CD@Fe‐700) and 5.5 wt.% (CD@Fe‐900). In addition, the hydrogen content gradually decreased from 1.22 wt.% (CD@Fe‐500) to 0.39 (CD@Fe‐700) and 0.23 wt.% (CD@Fe‐900). These resulted from the temperature‐dependent carbonization degree of Fe‐HBD precursor materials. While CD@Fe‐500 was in a state of incomplete carbonization, CD@Fe‐700 and CD@Fe‐900 were fully carbonized (Figure S2, Supporting Information). ICP‐AES analysis indicated that the Fe contents in CD@Fe‐500, CD@Fe‐700, and CD@Fe‐900 are 36.8, 42.5, and 45.6 wt.%, respectively.

**Figure 6:**
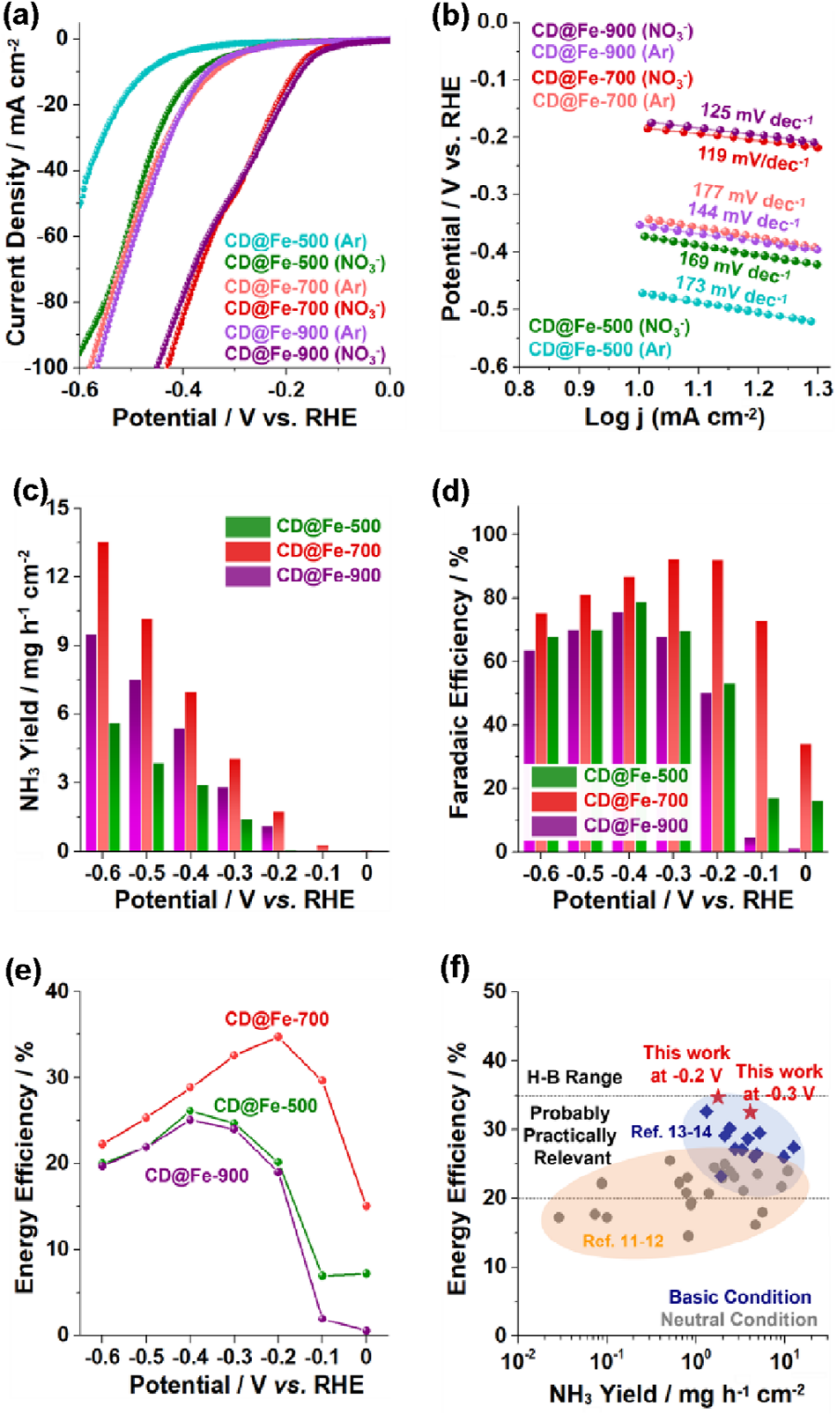
a) Linear sweep voltammetry curves and b) Tafel plots of the CD@Fe‐500–900 in basic condition (1 m KOH) with and without 0.1 m NaNO_3_. c) Ammonia yield rates, d) Faradaic efficiencies, and e) energy efficiencies of CD@Fe‐500–900 for electrochemical NO_3_RR in H‐type cells. f) Comparison of energy efficiencies of CD@Fe‐700 with the systems of literature^[^ ^11^, ^12^, ^13^, ^14^ ^]^ for NO_3_RR.

**Figure 7:**
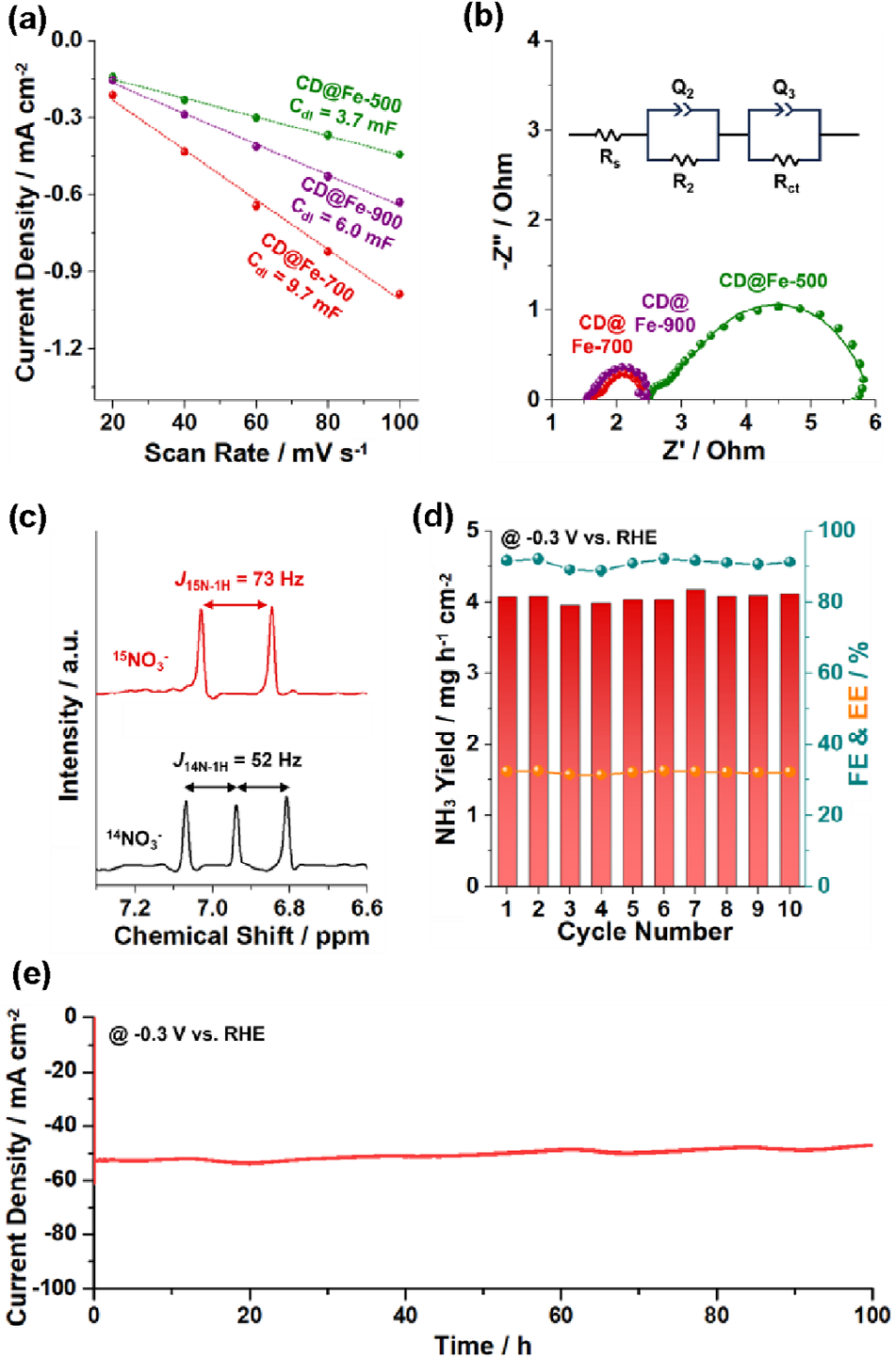
a) Electrochemical double‐layer capacitances (C_dl_) and b) Nyquist plots of CD@Fe‐500–900. c) ^1^H NMR spectra of ammonia solutions obtained by CD@Fe‐700 at −0.3 V (vs RHE) via an electrochemical ^15^NO_3_^−^ and ^14^NO_3_^−^ reduction reactions. d) Cyclability and e) long‐term stability tests of CD@Fe‐700 catalyst for the electrochemical NO_3_RR to ammonia at −0.3 V (vs RHE).

**Figure 8:**
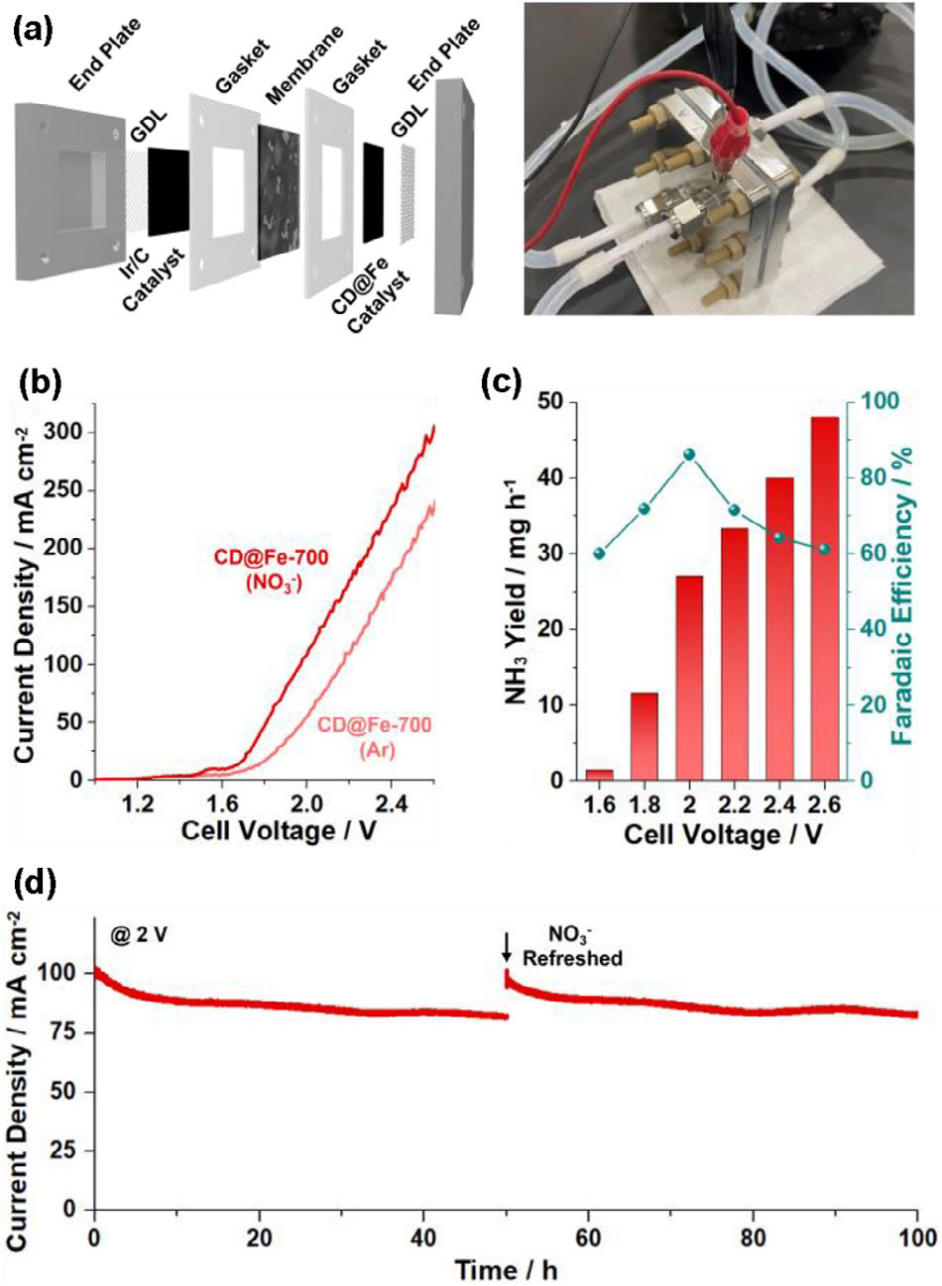
a) Structure and photograph of a zero‐gap cell used for NO_3_RR. b) LSV curves, c) cell voltage‐dependent ammonia yield rates and FEs, and d) long‐term stability tests (at a cell voltage of 2 V) of zero‐gap cells bearing CD@Fe‐700.

Considering that the original Haber‐Bosch process has utilized iron‐based catalysts, we studied the electrocatalytic performance of CD@Fe for the production of ammonia through the nitrate reduction reaction, denoted as a NO_3_RR process. The results in basic condition (1 Mm KOH) are summarized in Figures 6, 7, 8 (refer to Figure S6, Supporting Information for the results in neutral condition).

First, the NO_3_RR activities of CD@Fe electrocatalysts were evaluated using H‐type cells. The linear sweep voltammetry (LSV) curves demonstrated that current densities increased significantly in the presence of nitrate, compared to control reactions without nitrate, confirming the electrocatalytic activities of CD@Fe for NO_3_RR (**Figure** **6**a). The LSV studies indicated that CD@Fe‐700 and CD@Fe‐900 exhibited superior electrocatalytic activities to CD@Fe‐500. Tafel slopes were reduced in the presence of nitrate, compared to control tests without nitrate (Figure 6b). For example, the Tafel slope of CD@Fe‐700 was 177 mV/dec in the absence of nitrate, but decreased to 119 mV/dec in its presence, indicating facilitated redox processes (Figure 6b).

Chronoamperometric (CA) studies and UV/vis absorption‐based quantitative analysis of the ammonia generated by CD@Fe electrocatalysts were carried out (Figures S7 and S8, Supporting Information). The CA studies were performed at systematic potentials ranging from 0 to −0.60 V vs. reversible hydrogen electrode (RHE) for 0.5 h.

Among CD@Fe electrocatalysts, CD@Fe‐700 exhibited the best performance for NO_3_RR (Figure 6c). The ammonia yield rates of CD@Fe‐700 for NO_3_RR progressively increased from 1.76 mg h^−1^ cm^−2^ to 4.07 and 13.5 mg h^−1^ cm^−2^ with an increase of potentials from −0.20 V to −0.30 and −0.60 V (vs. RHE), respectively (Figure 6c). The corresponding Faradaic efficiencies (FEs) of CD@Fe‐700 gradually increased to 92.0 (at −0.20 V) and 92.3% (at −0.30 V) and then decreased to 75.4% (at −0.60 V), due to the competitive hydrogen evolution reaction (HER) (Figure 6d). The decreased FEs of CD@Fe‐900, compared to those of CD@Fe‐700 can be attributed to enhanced side reactions such as HER process facilitated by zerovalent Fe species. In situ potentiostatic Raman studies of the electrochemical process catalyzed by CD@Fe‐700 revealed the presence of NO_2_, NO, and NH_2_ intermediates^[^ ^26^ ^]^ on the surfaces at the potentials more negative than −0.10 V (vs. RHE), which correlated well with the potential‐dependent generation of ammonia (Figure S9, Supporting Information).

The energy efficiencies (EEs) of NO_3_RR catalyzed by CD@Fe were evaluated (Figure 6e). As potentials increased to −0.20 V (vs. RHE), the EEs of CD@Fe‐700 increased to 34.7%. Then, as potentials increased from −0.30 to −0.60 V, EEs gradually decreased from 32.6% to 22.2% (Figure 6e). The ammonia yield rates and EEs of CD@Fe‐700 are quite promising, compared to electrocatalytic systems reported in the literature (Figure 6f).^[^ ^11^, ^12^ ^]^ The promising performance of CD@Fe‐700 can be attributed to the well‐distributed Fe species in the carbon matrix, its surface‐rich disc morphology,^[^ ^27^ ^]^ high surface area, and meso/microporosity. It is noteworthy that the EEs of CD@Fe‐700 approached those (35–60%) of the Haber‐Bosch process (Figure 6f).

**Figures** **7**a and S10 (Supporting Information) show the double‐layer capacitances (C_dl_) of CD@Fe catalysts, which indicate the electrochemically active surface area (ECSA).^[^ ^11a^ ^]^ The C_dl_ values increased from 3.7 mF cm^−2^ (CD@Fe‐500) to 9.7 mF cm^−2^ (CD@Fe‐700) and then decreased to 6.0 mF cm^−2^ (CD@Fe‐900), corresponding to ECSAs of 93, 2.4 × 10^2^, and 1.5 × 10^2^ cm^2^, respectively (Figure 7a). Similarly, the diffusion coefficient increased from 0.42 × 10^−6^ cm^2^ s^−1^ (CD@Fe‐500) to 3.14 × 10^−6^ cm^2^ s^−1^ (CD@Fe‐700) and then decreased to 2.60 × 10^−6^ cm^2^ s^−1^ (CD@Fe‐900), matching well with surface areas and ECSAs of materials (Figure S11, Supporting Information).

Electrochemical impedance spectroscopy (EIS) showed that the charge transfer resistance (R_ct_) of CD@Fe‐700 was lowest (0.63 Ω), compared to those of CD@Fe‐500 (2.5 Ω) and CD@Fe‐900 (0.76 Ω), indicating the efficient distribution of Fe species in the conductive carbon matrix of CD@Fe‐700 (Figure 7b; Figure S12, Supporting Information). Moreover, in situ potentiostatic EIS studies showed that as potentials increased from 0 to −0.7 V, R_ct_ gradually decreased (Figure S13, Supporting Information). Overall, R_ct_ and solution resistance (R_s_) decreased in the order of CD@Fe‐500 > CD@Fe‐900 > CD@Fe‐700 (Figure S13, Supporting Information). In addition, R_ct_ decreased more significantly at higher potentials under NO_3_RR conditions, compared to those under HER conditions, indicating that NO_3_RR is more facile than HER process (Figure S14, Supporting Information).

Isotope labeling studies were conducted using ^15^NO_3_^−^ and control ^14^NO_3_^−^ as nitrogen sources for NO_3_RR. The ^1^H NMR spectrum of ammonia solution generated by CD@Fe‐700 with ^15^NO_3_^−^ showed a doublet peak with a coupling constant of *J_15N‐1H_* = 73 Hz, corresponding to ^15^NH_4_^+^ (Figure 7c).^[^ ^11b,c^ ^]^ It is noteworthy that conventional ^14^NH_4_^+^ shows a triplet peak with a coupling constant of *J_14N‐1H_* = 52 Hz.^[^ ^11b,c^ ^]^ Thus, ^1^H NMR studies confirmed that the produced ammonia originated from the reduction of the supplied nitrate and not from impurities containing ^14^N species.

The stability of CD@Fe‐700 for NO_3_RR was evaluated by cycling and long‐term chronoamperometry tests at −0.30 V (vs. RHE). No significant changes were observed in the ammonia yield rates, FEs, and EEs during consecutive 10 cycles (Figure 7d; Figure S15, Supporting Information). The chronoamperometry test over continuous electrochemical reactions for 100 h displayed a steady current density, further attesting to the long‐term durability of CD@Fe‐700 (Figure 7e). It is noteworthy that during the electrochemical reactions, the pH of the electrolyte was maintained within the range of 14.0–14.1. TEM and EDS‐based elemental mapping analysis of the recovered CD@Fe‐700 catalysts indicated retention of the original morphology and chemical structure (Figure S16, Supporting Information). In addition, while the molar ratio of zerovalent Fe to cationic Fe species measured by ex situ XPS studies changed to 1.9:98.1 under basic electrolyte conditions, it was largely preserved at 1.6:98.4 after 100 h of electrochemical reactions (Figure S17, Supporting Information).

Next, ammonia production through CD@Fe‐700‐based NO_3_RR was studied in a zero‐gap cell (**Figure** **8**; Figures S18 and S19, Supporting Information). Generally, zero‐gap cells have advantages over H‐type cells in terms of lower ohmic and mass transport losses (Figure S18, Supporting Information). In this regard, the zero‐gap cells were fabricated using end plates, Ni foam‐based gas diffusion layers (GDLs), gaskets, a CD@Fe‐700 cathode as the reduction catalyst, an Ir/C anode as the oxidation catalyst, a Nafion 117 membrane separator, and 1 m KOH solution as electrolyte (Figure 8a). While the nitrate solution was supplied into zero‐gap cells, the ammonia solution was retrieved. The LSV curves indicated that onset potentials decreased in the presence of nitrate, indicating that the NO_3_RR process successfully worked (Figure 8b). As the cell voltages increased from 1.6 V to 1.8, 2.0, 2.2, 2.4, and 2.6 V, the ammonia yield rates gradually increased from 1.47 mg h^−1^ to 11.7, 27.1, 33.4, 40.1, 48.1 mg h^−1^, respectively (Figure 8c; Figures S19, Supporting Information). The corresponding FEs increased from 60.0% (1.6 V) to 71.8% (1.8 V), peaked at 86.2% (2.0 V), and then gradually declined to 71.5 (2.2 V), 64.2 (2.4 V), and 61.1% (2.6 V).

Long‐term stability tests of the zero‐gap cell fabricated with CD@Fe‐700 were conducted for NO_3_RR at a cell voltage of 2.0 V (Figure 8d). The current densities (j) were retained for 100 h with a single supply of fresh 0.1 M NaNO_3_ solution at 50 h, showing the j_55 h_/j_5 h_ of 0.997 and the j_100 h_/j_50 h_ of 1.006.

Next, to elucidate the electrocatalytic roles of zerovalent Fe and cationic Fe species in Fe_3_O_4_, we conducted computational studies based on the density functional theory (DFT) method (**Figure** **9**; Figures S20–S26 and Table S3, Supporting Information for details).

**Figure 9:**
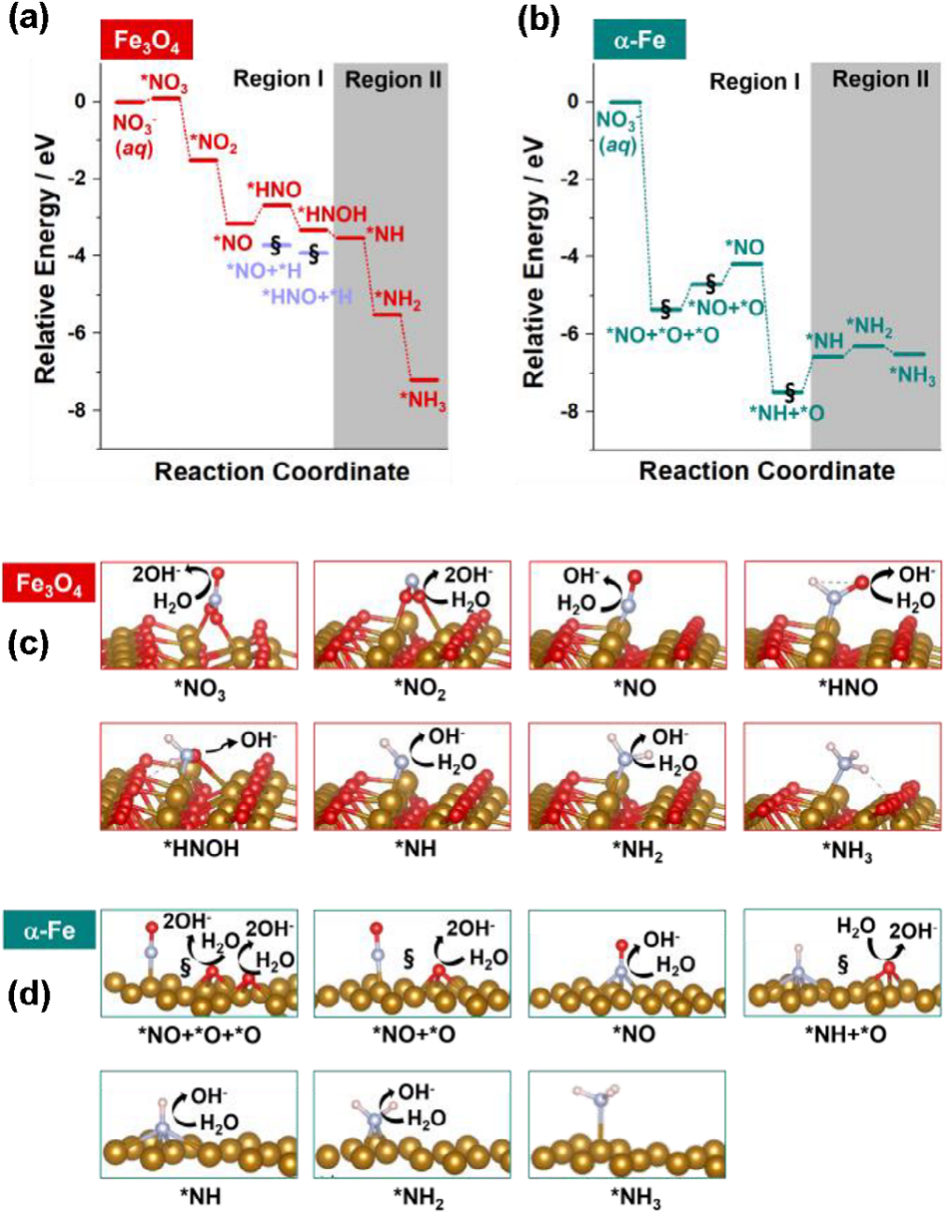
Energy profiles and intermediate structures for the reduction of NO_3_^−^ in solution to form *NH_3_ on a,c) Fe_3_O_4_ (311) and b,d) α‐Fe (110) surfaces predicted by DFT calculations. The § notations represent the spontaneous dissociation reactions.

The predicted minimum energy pathways for the reduction of nitrate to NH_3_ on Fe_3_O_4_ (311) and α‐Fe (110) surfaces based on electronic energies are shown in Figure 9a,b, respectively, and Table S3 (Supporting Information). The optimized structures for all intermediates are shown in Figure 9c,d. Herein, the Regions I and II, marked by white and gray regions, correspond to before and after *NH formation, respectively. In Region I, on the Fe_3_O_4_ surface, it is expected that the reduction process from *NO_3_ to *NH occurs via stepwise reactions: *NO_3_ → *NO_2_ → *NO → *HNO → *HNOH → *NH (Figure 9a,c; Figures S21 and S22, Supporting Information). In alkaline solutions, the active hydrogens are generated from the water dissociation reaction (Figure 9c). It is worth noting that other intermediates having the lowest energy (sky blue lines with § notations) were also found from *NOH and *HNOH conformers (Figure 9a; Figure S23, Supporting Information). However, during geometrical optimization steps, the O─H bonds in *NOH and *HNOH conformers were spontaneously cleaved to form *NO + *H and *HNO + *H, respectively. (Movie S1 and S2, Supporting Information) The dissociated H fragment makes a new chemical bond with the O atom exposed on the Fe_3_O_4_ surface, which indicates the reduction of the Fe_3_O_4_ surface. Since the dissociated *NO and *HNO fragments are identical to the former intermediates, these O─H dissociation reactions could suppress the further reduction process of nitrogen species. For this reason, as a bypass, the reduction reactions of NO and HNO intermediates inevitably could occur toward *HNO and *HNOH having higher energies than *NO + *H and *HNO + *H by 1.05 and 0.59 eV, respectively, on the Fe_3_O_4_ surface.

On the other hand, our DFT calculations show that α‐Fe does not exhibit such surface reduction reactions (Figure 9b,d; Figures S24–S26, Supporting Information). Instead, the spontaneous deoxygenation reactions mainly can lead to the reduction process of nitrogen species on α‐Fe: *NO_3_ → *NO + *O + *O and *HNO → *NH + *O (Figure 9b,d; Figure S26, and Movies S3 and S4, Supporting Information). The dissociated O fragment forms the partially oxidized α‐Fe surface. *NH formation starting from NO_3_^−^(*aq*) on α‐Fe is energetically favorable by ‐6.58 eV, indicating the energy release in an exothermic reaction. This value is much higher than that of Fe_3_O_4_ (−3.53 eV). Therefore, the energy profiles shown in Region I imply that *NH formation on α‐Fe can be more effective than on Fe_3_O_4_ due to energetic favorability through the deoxygenation reaction pathway.

After the *NH shown in Region II, Fe_3_O_4_ and α‐Fe follow the same reaction pathways consisting of stepwise reactions: *NH → *NH_2_ → *NH_3_ (Figure 9a–d; Table S3, Supporting Information). However, it is expected that Fe_3_O_4_ and α‐Fe have remarkably different energy trends. Fe_3_O_4_ shows the energetically downhill trend, which can give the advantage of electronic energy for facilitating the progressive hydrogenation reactions of *NH to form *NH_3_ (Figure 9a). By contrast, α‐Fe exhibits the endothermic tendency (Figure 9b). Therefore, Fe_3_O_4_ might become a better catalyst in Region II compared to α‐Fe, because the electronic energy changes for the reaction *NH → *NH_3_ are −3.67 and +0.07 eV for Fe_3_O_4_ and α‐Fe, respectively.

As a result, the energy profiles in Figure 9 reveal that α‐Fe and Fe_3_O_4_ can play a significant role in the nitrate reduction reactions before and after the *NH intermediate (Region I and II), respectively. Thus, the heterostructure catalyst composed of both α‐Fe and Fe_3_O_4_ provides a necessary condition to efficiently enhance the production of NH_3_, even though the *NH intermediate generated on α‐Fe can be transferred to Fe_3_O_4_ over their heterogeneous interface. The energy required to migrate *NH on the α‐Fe surface to the Fe_3_O_4_ surface is estimated to be +3.05 eV, which is sufficiently compensated by the energy for the subsequent exothermic hydrogenation of *NH to *NH_3_ on Fe_3_O_4_ (−3.67 eV). In this regard, our DFT results are in line with higher NH_3_ yield/Faradaic efficiency for CD@Fe‐700 and CD@Fe‐900, composed of both α‐Fe and Fe_3_O_4_, compared to CD@Fe‐500, which is only composed of Fe_3_O_4_. The best catalytic performance of CD@Fe‐700 might be related to the optimal ratio of α‐Fe/Fe_3_O_4_ for catalyst optimization. While the catalytic roles of α‐Fe and Fe_3_O_4_ were predicted by the calculation of electronic energies, the effect of Gibbs free energy terms on the various reaction pathways will be investigated in future work.

## 3 Conclusion

This work shows that iron‐based electrocatalysts were prepared for the nitrate reduction to ammonia. The morphology of CD@Fe could be engineered by the formation of disc‐shaped precursor materials through the reaction of iron ions with 2,5‐dihydroxy‐1,4‐benzoquinone. The pyrolysis of Fe‐HBD precursors resulted in the formation of iron nanoparticles homogeneously distributed on the carbon disc matrix. As the heating temperature increased from 500 to 700 °C and 900 °C, the contents of zerovalent iron increased with an increase of particle sizes. The optimal CD@Fe‐700 with a high surface area of 424 m^2^ g^−1^ and micro/mesoporosity showed excellent performance in the electrocatalytic nitrate reduction to ammonia, showing ammonia yield rates up to 1.8∼13.5 mg h^−1^ cm^−2^ at the overpotentials of −0.20–−0.60 V (vs. RHE). The energy efficiencies (32.6–34.7%) of CD@Fe‐700‐based NO_3_RR processes reached those of the Haber‐Bosch process. The promising efficiency of CD@Fe‐700‐based electrocatalytic system is attributable to the homogeneously distributed iron nanoparticles, the surface‐rich disc shape, a high surface area, and micro/mesoporosity, enabling interaction with nitrate. We suggest that the electrocatalytic performance of CD@Fe can be further improved through optimization of morphology parameters and doping of secondary metals.^[^ ^12^, ^14^ ^]^

## Conflict of Interest

The authors declare no conflict of interest.

## Supporting information



Supporting Information

Supplemental Movie 1

Supplemental Movie 2

Supplemental Movie 3

Supplemental Movie 4

## Data Availability

The data that support the findings of this study are available in the supplementary material of this article.
