# Supplementary material for: Iron Nanoparticles on Porous Carbon Discs: Electrocatalysts with Over 30% Energy Efficiencies for the Production of Ammonia from Nitrate
Source: Adv Sci (Weinh). 2025 Sep 29;12(47):e14504. doi: 10.1002/advs.202514504 (PMC12713075; doi:10.1002/advs.202514504)
Supplement: Supplementary file 1 — Supporting Information [file ADVS-12-e14504-s003.pdf]

Copyright WILEY-VCH Verlag GmbH & Co. KGaA, 69469 Weinheim, Germany, 2025.

Supporting Information

Iron Nanoparticles on Porous Carbon Discs: Electrocatalysts with Over 30% Energy Efficiencies for the Production of Ammonia from Nitrate

Daon Park,[a]† Jinuk Choi,[b]† Ji Yong Shim,[c]† Yoon Kee Kim,[a] Jina Park,[a] Chang Wan Kang,[a] So Young Park,[a] Tae-Yong An,[b] Hyojung Lim,[b] Subramani Surendran,[b] Kyoung Chul Ko,[c] Uk Sim,[b,d]* and Seung Uk Son[a]**

Experimental

General Information: SEM studies were performed using a JSM-7100F instrument at the Chiral Material Core Facility Center of Sungkyunkwan University. TEM, HR-TEM, and EDS-based elemental mapping studies were conducted using a JEOL-2100F instrument. IR studies were performed using a Bruker VERTEX 70 FT-IR spectrometer at the Chiral Material Core Facility Center of Sungkyunkwan University. ^{1}H NMR studies were conducted a Bruker Avance III 400 MHz instrument. TGA curves were obtained using a Seiko Exstar 7300 instrument. Surface area and porosity of materials were analyzed using a Micromeritics ASAP2020 equipment. Pore size distribution diagrams were obtained by the NLDFT method. PXRD studies were conducted using a Rigaku MAX-2200 instrument. XPS spectra were obtained using a Thermo VG spectrometer. Combustion-based elemental analysis was conducted using a FLASH2000 instrument. ICP-AES analysis was conducted using an OPTIMA 8300 equipment. Electrochemical studies were performed using an electrochemical workstation (Biologic VSP 200, France). In-situ Raman measurements were carried out in a three-electrode configuration using a Raman spectrometer (inVia Qontor, Renishaw, U.K.) in 1 M KOH containing 0.1 M $NaNO_3$. Spectra were acquired at open circuit potential (OCP) and at various applied potentials ranging from 0.2 to -0.5 V *vs.* RHE. Electrochemical impedance spectroscopy (EIS) was performed in 1 M KOH with and without 0.1 M $NaNO_3$ over a frequency range of 10 kHz to 100 mHz, at applied potentials ranging from 0 to -0.7 V *vs.* RHE.

Synthesis of Fe-HBD

For the preparation of iron(III) acetate,[1] anhydrous iron(II) acetate (50 g, 0.29 mol) was dissolved in acetic acid aqueous solution (50%, 18 mL) by stirring at 75 ºC for 30 min. After H_2O_2 (8 mL) was added dropwise, the reaction mixture was stirred for 3 h. During this process, gas evolved. After cooling to room temperature, the reaction mixture was filtered through Celite to remove black impurities. After the filtered solution was evaporated, the dark red residues (iron(III) acetate) were dried under vacuum.

For the preparation of Fe-HBD, iron(III) acetate (82 mg, 0.35 mmol) was dissolved in DMF (6 mL) in a 10 mL vial. After 2,5-dihydroxy-1,4-benzoquinone (0.10 g, 0.71 mmol) dissolved in DMF (5 mL) was added, the reaction mixture was sonicated for 30 min. After the vial was sealed with a cap, the reaction mixture was

treated at 80 ºC for 2 h without stirring in an oven. After cooling to room temperature, the precipitates (Fe-HBD) were separated by centrifugation, washed with diethylether (10 mL) once and a mixture of methanol (10 mL) and diethylether (10 mL) three times, and dried under vacuum.

Synthesis of CD@Fe

After Fe-HBD (0.20 g) was loaded into an alumina ceramic crucible boat, it was heated at 500, 700, or 900 ºC at the heating rates of 20 ºC/min for 3 h under argon. The resulting materials were denoted as CD@Fe-500, CD@Fe-700, and CD@Fe-900, respectively. After cooling to room temperature, the CD@Fe was collected. The surface of iron nanoparticles in CD@Fe was naturally oxidized under air. Caution: CD@Fe-500 can especially cause fire during air oxidation, due to the generation of fine zerovalent Fe nanoparticles in carbon matrixes.

Procedures of electrocatalytic studies

The electrochemical NO_3RR activity of CD@Fe catalysts was evaluated using an H-type cell (StonyLab H-type Electrolytic Cell, U. S.) with a three-electrode system controlled by an electrochemical workstation (Biologic VSP 200, France). For all electrocatalytic studies, a Hg/HgO electrode and a platinum sheet electrode (1 cm × 1 cm) were employed as reference and counter electrodes, respectively. A 1 M KOH solution (50 mL, pH 14) with or without 0.1 M $NaNO_3$ was used as an electrolyte. A proton exchange membrane (Nafion 117) was used to separate the cathode from the anode. CD@Fe catalysts (5.0 mg) were dispersed in 450 μL of ethanol, followed by the addition of 50 μL of Nafion solution (Sigma-Aldrich, 20 wt.%) to make a homogeneous solution (10 mg mL^{-1}). The solution was ultrasonicated for about 0.5 h to ensure that good dispersion of catalyst inks. Afterward, catalyst inks (90 μL) were dropped onto the carbon cloth (CC) with an area of 1 cm × 1 cm and dried overnight at 80 ºC in a vacuum oven. The loading amount of CD@Fe catalysts was calculated to be 1.5 mg cm^{-2}. The potentials reported in this study were converted to a RHE scale by a calibration procedure using the following equation: E (V vs. RHE) = E (V vs. Hg/HgO) + 0.118 + 0.0592 × pH.[2] The theoretical potential for NO_3RR is 0.69 V vs. RHE. All NO_3RR performances of CD@Fe catalysts were measured under ambient conditions (1 atm, 25 °C). Ar gas was continuously pumped into the cathode chamber for 30 minutes before the tests to eliminate the influence of impurity gases. The linear sweep voltammetry (LSV) measurements were performed at a scan rate of 5 mV s^{-1} with 85% IR-compensation of potential. Chronoamperometry (CA) measurements were conducted for 0.5 h at selected potentials ranging from 0.0 to -0.6 V *vs.* RHE. The electrochemical surface area (ECSA) was calculated as follows: ECSA = C_{dl}/C_s and $C_{dl} = i_c/v$ where C_s is the specific capacitance (C_s = 0.040 mF cm^{-2}), i_c is the current density (mA cm^{-2}), and v is the scan rate (mV s^{-1}). The cyclic voltammetry (CV) curves used to determine C_{dl} were measured within a potential window where nonfaradaic current was observed. These measurements were conducted in 1.0 M KOH electrolyte with incremental scan rates of 20, 40, 60, 80, and

100 mV s^{-1}, respectively. The electrochemical impedance spectroscopy (EIS) measurements were conducted under the same working conditions as the previous tests, with a frequency range from 100 kHz to 0.01 Hz at -0.3 V *vs.* RHE. During the stability test, the catholyte (0.1 M $NaNO_3$ + 1 M KOH) was continuously recirculated using a digital peristaltic pump (EMS Tech EMP-600A, Rep. of Korea) at a flow rate of 5 mL min^{-1} to maintain the NO_3^- concentration over 100 hours.

Additionally, the NO_3RR performances of CD@Fe-700 were evaluated in a custom-built zero-gap cell (2 cm × 2 cm, NEEL Sci., Rep. of Korea) with a flow channel setup. The cell was assembled with integrated end plates, porous transport layers (PTLs), an anode, a cathode, gaskets, and a membrane (Nafion 117), as shown in Figure 8a. CD@Fe-700 on CC (2 × 2 cm^2) and Ir/C (99.8%, metals basis, Alfa Aesar, U.S.) on nickel foam (2 × 2 cm^2) were used as the cathode and anode, respectively. The flow rate of the electrolytes on both the anode and cathode sides was maintained at 50 mL min^{-1} using a digital peristaltic pump during electrochemical tests. The anolyte was 1 M KOH solution, and the catholyte was 0.1 M $NaNO_3$ + 1 M KOH solution. The LSV measurements were performed at a scan rate of 10 mV s^{-1} without IR-compensation. CA measurements were conducted for 0.5 h at selected potentials ranging from 1.6 to 2.6 V. During the stability test, the electrolytes (5 L) were refreshed after 50 hours to sustain the NO_3^- concentration over 100 hours.

Procedures of additional electrocatalytic studies under neutral conditions

The electrochemical NO_3RR activities of CD@Fe catalysts were evaluated under neutral conditions. All testing parameters were the same as those in the previous test (conducted under alkaline conditions), except for the electrolyte condition.

For the NO_3RR tests under neutral conditions, 0.25 M Na_2SO_4 + 0.5 M $NaNO_3$ was used as the catholyte, and 0.25 M Na_2SO_4 was used as the anolyte. CA measurements were conducted for 1 h at selected potentials ranging from -0.35 to -0.85 V *vs.* RHE. CV measurements for ECSA calculation were conducted in 0.25 M Na_2SO_4 electrolyte with incremental scan rates of 20, 40, 60, 80, and 100 mV s^{-1}, respectively. EIS measurements were conducted under the same working conditions as the previous tests at -0.65 V *vs.* RHE.

Quantification procedures of ammonia

The produced ammonia was quantified using a colorimetric indophenol blue method[3] for three separate experiments with different catalyst samples. The presented ammonia production rate is the average rate of three different experiments. First, after the chronoamperometry test, the electrolyte (2 mL) was collected. Then, solution (1.25 mL) containing 0.625 M NaOH, 0.36 M salicylic acid and 0.17 M sodium citrate was added. After sodium nitroferricyanide solution (10 mg mL^{-1}, 150 μL) and NaClO solution (10~15 wt%, 75 μL) were added, the mixture was left standing at 25 °C for 30 min, generating a blue-colored reactant. The concentration of NH_3 in the electrolyte was determined through UV-vis absorption spectroscopy (Thermo Fisher Evolution One Plus, U. S.) based on absorbance values at 650 nm. The absorbance versus

concentration was calibrated using standard NH_4Cl with a 0.1 M $NaNO_3$ + 1 M KOH electrolyte and a series of NH_3/NH_4^+ concentrations (0, 0.5, 1, 2, and 4 μg mL^{-1}). The fitting curve (y = 0.1372*x + 0.0008215, R^2=0.99993) shows a linear relationship between absorbance and ammonia concentration. Additionally, the produced NH_3 under neutral conditions was also quantified using a colorimetric indophenol blue method. The concentration of NH_3 in the electrolytes was determined through UV-vis absorption spectroscopy based on absorbance values at 680 nm. The absorbance versus concentration was calibrated using standard NH_4Cl with a 0.5 M $NaNO_3$ + 0.25 M Na_2SO_4 electrolyte and a series of NH_3/NH_4^+ concentrations (0, 0.5, 1, 2, and 4 μg mL^{-1}).

Procedures of isotope-labeled experiments

$Na^{15}NO_3$ was utilized as the nitrogen feeding source for the isotopic labeling of nitrate reduction, verifying the origin of ammonia. Before performing ^1H NMR (400 MHz, Bruker Avance III HD 400, U. S.) measurements, the NO_3RR test was conducted for 1 h at -0.3 V *vs.* RHE to produce $^{15}NH_4^+$ using $Na^{15}NO_3$. The collected electrolyte was acidified to pH 2 using 1 M HCl. The solution (0.5 mL) was mixed with DMSO-d_6 (Sigma-Aldrich, 99.9%, 0.1 mL). Similarly, $^{14}NH_4^+$ was detected by the same method when $Na^{14}NO_3$ was used as the nitrogen source.

Determination procedures of yields and Faradaic efficiency (FE)

The yield rate of NH_3 (R_{NH3}) was calculated as follows: $R_{NH3} = [NH_3] \times V/(A \times t)$.[4] Faradaic efficiency (FE) was calculated based on the charge consumed for synthesized ammonia and the total charge passed through the electrode, as follows: $FE(\%) = (n\ e^-) \times F \times [NH_3] \times V/(17 \times |Q|) \times 100$, where $[NH_3]$ is the measured concentration of NH_3 (μg mL^{-1}) in the electrolyte, V is the electrolyte volume, A is the geometric electrode area (1 cm × 1 cm), t is the electrolysis time, n is the number of electrons, F is the Faraday constant (96485 C mol^{-1}), and Q is the total charge passed through the electrode. The chemical equation used is as follows: NO_3RR in basic solution: $NO_3^-(aq) + 6H_2O + 8e^- \rightleftarrows NH_3(aq) + 9OH^-(aq)$, n of e^- = 8.

Determination procedures of energy efficiency (EE)

The half-cell energy efficiency (EE) of NH_3 production is defined as the ratio of fuel energy to applied electrical power. EE is calculated as the product of Faraday efficiency for NH_3 (FE_{NH3}) and voltage efficiency (VE), as follows: $EE(\%) = FE_{NH3} \times VE = FE_{NH3} \times (E^0_{OER} - E^0_{NH3})/(E^0_{OER} – E_{NH3})$, where E^0_{OER} and E^0_{NH3} are the equilibrium potentials of oxygen evolution reaction (OER, 1.23 V *vs.* RHE) and nitrate reduction reaction (NO_3RR, 0.69 V *vs.* RHE), respectively, and E_{NH3} is the applied potential.

Procedures of computational studies

To understand the reason for the difference in catalytic activities between substances (CD@Fe-500, CD@Fe-700, and CD@Fe-900) having different Fe(0) contents in Fe_3O_4 based on reaction mechanisms, the density functional theory (DFT) calculations were carried out for both α-Fe and Fe_3O_4 substrates. In our DFT study, the Perdew–Burke–Ernzerhof (PBE) functional/light-tier-1 level of theory was used. The convergence criterion was set to 0.01 eV/Å. The zeroth-order regular approximation (ZORA) was used to incorporate the relativistic effects for heavy metal atoms. To design a slab model, single unit cells of Fe_3O_4 (*Fd-3m* space group) and α-Fe (*Im-3m* space group) provided by the American Mineralogist Crystal Structure Database (AMCSD)[5] were optimized with full relaxation of atomic positions and lattice parameters using 7×7×7 k-point mesh. To properly describe the electronic structures of ferrimagnetic Fe_3O_4 and ferromagnetic α-Fe, we intended to assign the initial atomic charges and spins for Fe^{2+}, Fe^{3+}, and Fe atoms by referring to previous theoretical studies for inverse spinel Fe_3O_4 and bcc iron.[6,7] As a reference plane, we selected the (311) facet and (110) facet, which are clearly observed by HR-TEM experiments (Figure 4a–c in text) for Fe_3O_4 and α-Fe, respectively. The Fe_3O_4 (311) and α-Fe (110) surfaces are generated by cleavage of their optimized single-unit cells along a crystallographic plane. To avoid spurious interactions between the slabs themselves, we added vacuum spaces 20.0 Å along the z-axis for Fe_3O_4 and α-Fe surfaces, respectively. The designed Fe_3O_4 (311) surface slab model contains 112 atoms with the lattice parameters of a = 10.28 Å, b = 11.87 Å, c = 29.53 Å, α = 90.0, β = 90.0, and γ = 73.22. The α-Fe (110) surface slab model comprises 108 atoms having a = 8.50 Å, b = 12.02 Å, c = 26.01 Å, α = 90.0, β = 90.0, and γ = 90.0. For surface slab model calculations, the geometric optimizations were conducted only for atomic positions, while a half of the layers and the lattice parameters were fixed. A 3×3×1 Monkhorst–Pack k-point mesh was used. All DFT calculations were carried out using the FHI-aims code.[8]

Using total SCF (Self-consistent field) energy of HNO_3, $4H_2$, and a support as the reference state, the relative energy for the nitrate ions in solution ($NO_3^-(aq)$) was estimated from the experimental formation enthalpies for $HNO_3(g)$[9] and $NO_3^-(aq)$[10] at the temperature of 298 K. Based on the enthalpy change for $NO_3^-\ (aq) + H^+\ (aq) \rightarrow HNO_3\ (g)$, we applied the assumption of an ideal gas ($PV = nRT$) to approximately compute the internal energy of $NO_3^-(aq)$. Then, the electronic energy of $NO_3^-(aq)$ was estimated as the internal energy, because the zero-point energy contribution and thermal correction are much smaller than that of electronic and solvation energies. Finally, the total electronic energy of $NO_3^-(aq)$, $4H_2$, and a substrate was set to zero and we drew the energy profile for the relative energies of the intermediates adsorbed on the substrate (denoted as *A).

To predict the energy pathways for the reduction reactions of NO_3^- to produce NH_3, we searched for the stable intermediates by the following ways: (1) Defining the various adsorption sites on a surface slab model, (2) Finding the most stable adsorption structure on the adsorption sites with consideration of the number of cases for binding structures, (3) Determining the most stable intermediates along with the consecutive

reaction pathway from the former step. The Figures S13a and S13b in the SI show the considered adsorption sites on Fe_3O_4 (311), and α-Fe (110), respectively. The binding structures and their relative energies for the optimized NO_3 and NO_2 adsorbed on various sites of substrates (*NO_3 and *NO_2) were depicted in Figures S14, S15, S17 and S18 in the SI, respectively. For later reaction steps, we assumed that the reactions occur consecutively starting from the most stable intermediate in the previous step. We considered possible cases for reduction reactions with an activated hydrogen, and finally selected the most stable solution. The optimized geometries for the intermediates and their relative energies in the energy profile were shown in Figure 9c-d and Table S3 in the SI, respectively.

Reference

[1] A. Laurikenas, J. Barkauskas, J. Reklaitis, G. Niaura, D. Baltrunas, A. Kareiva, *Lith. J. Phys.* **2016**, *56*, 35-41.

[2] C.-T. Hsieh, X.-F. Chuah, C.-L. Huang, H.-W. Lin, Y.-A. Chen, S.-Y. Lu, *Small Methods* **2019**, *3*, 1900234

[3] Y. Liu, L. Wang, L. Chen, H. Wang, A. R. Jadhav, T. Yang, Y. Wang, J. Zhang, A. Kumar, J. Lee, V. Q. Bui, M. G. Kim, H. Lee, *Angew. Chem. Int. Ed.* **2022**, *61*, *e202209555.*

[4] a) J. Wang, L. Yu, L. Hu, G. Chen, H. Xin, X. Feng, *Nat. Commun*. **2018**, *9*, 1795. b) S. E. Braley, J. Xie, Y. Losovyj, J. M. Smith, *J. Am. Chem. Soc.* **2021**, *143*, 7203-7208.

[5] The webpage address of the American Mineralogist Crystal Structure Database (AMCSD) is http://rruff.geo.arizona.edu/AMS/amcsd.php

[6] J. P. Attfield, *APL Mater.* **2015**, *3.*

[7] D. Dragoni, T. D. Daff, G. Csányi, N. Marzari, *Phys. Rev. Mater.* **2018**, *2.*

[8] V. Blum, R. Gehrke, F. Hanke, P. Havu, V. Havu, X. Ren, K. Reuter, M. Scheffler, *Comput. Phys. Commun.* **2009**, *180*, 2175-2196.

[9] C. MW Jr, *J. Phys. Chem. Ref. Data, Monograph*, **1998**, *9*, 1-1951.

[10] B. Ruscic, D. Bross, *Active Thermochemical Tables (ATcT) values based on ver. 1.122 of the Thermochemical Network*, **2016**, available at ATcT. anl. gov.

Figure S1. TGA curves of Fe-HBD and CD@Fe.

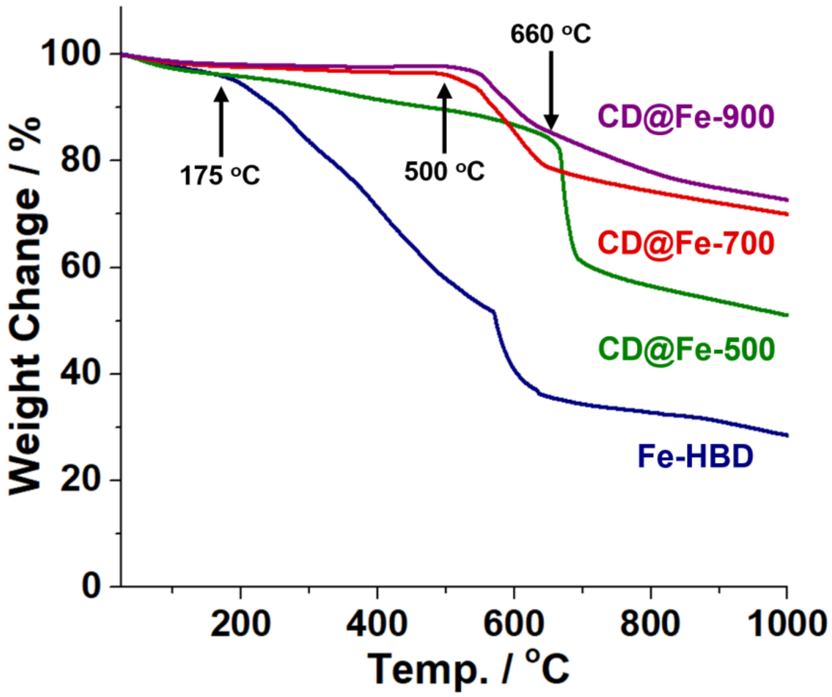

Figure S2. IR absorption spectra of Fe-HBD and CD@Fe.

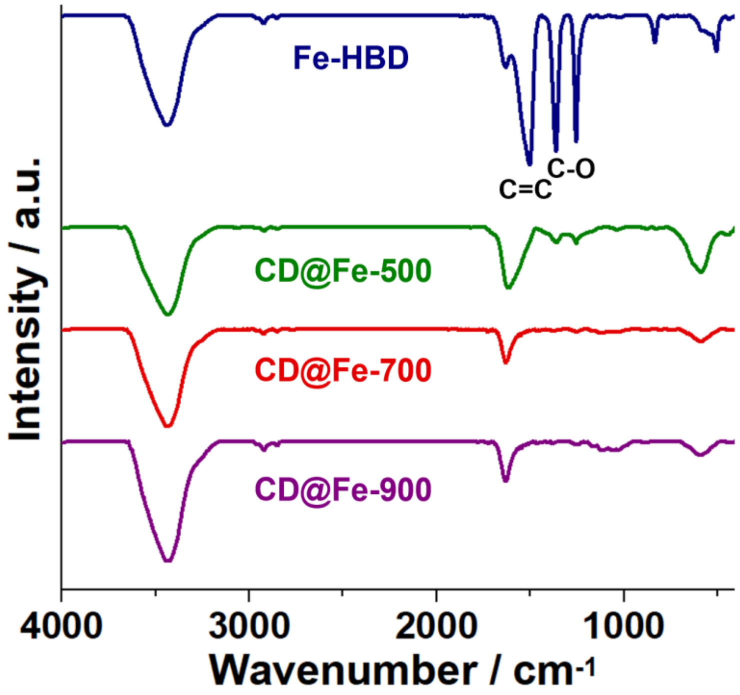

Figure S3. EDS-elemental mapping TEM images of Fe-HBD.

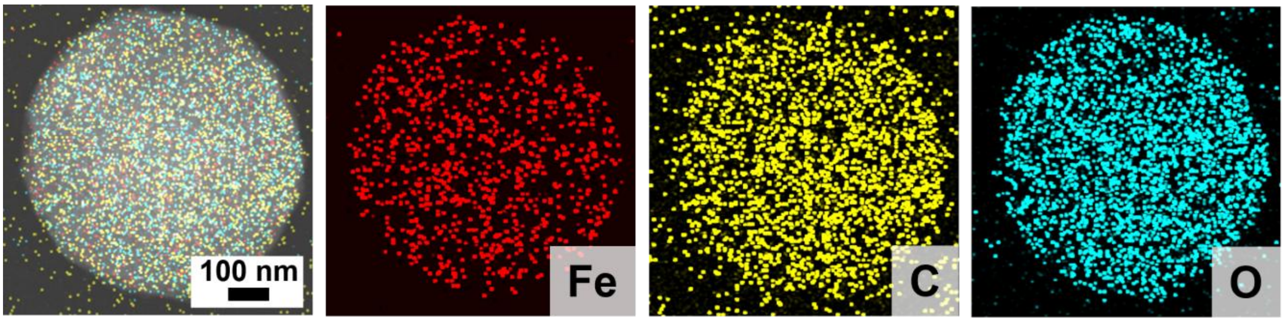

Figure S4. TEM images of carbon matrixes obtained from iron etching from (a) CD@Fe-500, (b) CD@Fe-700, and (c) CD@Fe-900.

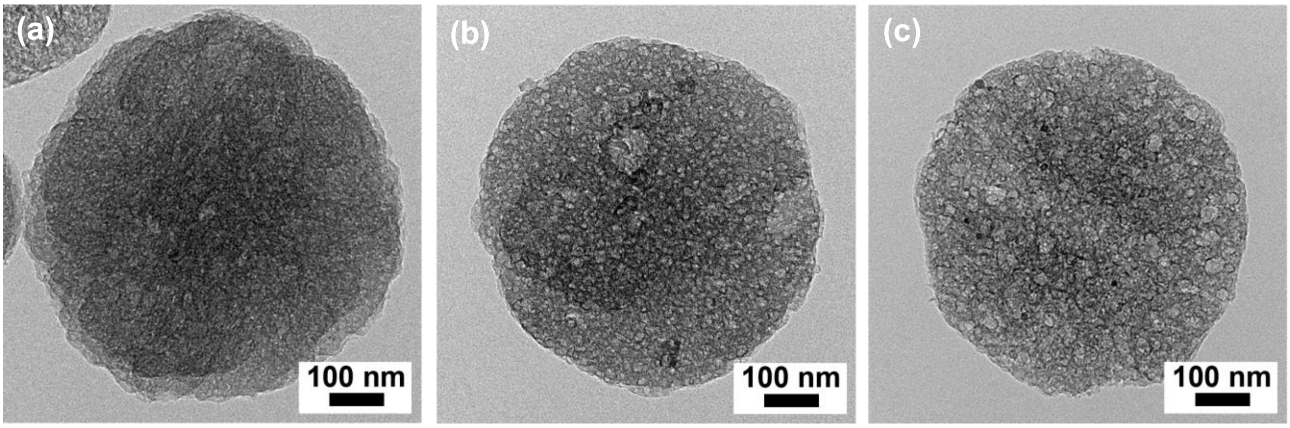

Figure S5. Analysis of XPS (a) Fe 2p, (b) O 1s, and (c) C 1s orbital spectra of Fe-HBD and CD@Fe.

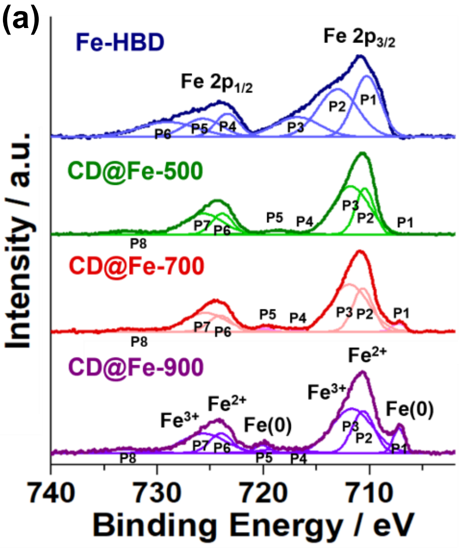

	Peak	P1	P2	P3	P4	P5	P6
Fe-HBD	Position (eV)	710.26	712.98	716.77	723.55	726.63	730.85
	FWHM	2.98	4.33	4.79	3.04	4.87	5.43
	Area (%)	24.8	28.5	13.5	11.0	15.4	6.73
	r^2	0.994					

	Peak	P1	P2	P3	P4	P5	P6	P7	P8
CD@Fe -500	Position (eV)	-	710.46	711.78	718.76	-	723.87	725.64	732.81
	FWHM	-	2.00	3.98	4.03	-	2.29	4.29	3.34
	Area (%)	-	20.8	43.1	3.43	-	10.7	20.1	1.97
	r^2	0.997							
CD@Fe -700	Peak	P1	P2	P3	P4	P5	P6	P7	P8
	Position (eV)	707.28	710.64	711.89	718.73	720.05	724.06	725.46	732.34
	FWHM	1.45	2.23	4.16	2.68	1.67	2.56	4.52	3.18
	Area (%)	2.61	21.2	43.3	2.46	1.17	8.99	18.8	1.53
	r^2	0.997							
CD@Fe -900	Peak	P1	P2	P3	P4	P5	P6	P7	P8
	Position (eV)	707.23	710.60	711.67	718.26	720.10	723.98	725.66	733.09
	FWHM	1.24	2.42	4.49	4.24	1.61	2.51	4.86	3.82
	Area (%)	5.90	19.7	38.4	3.34	2.38	9.47	18.2	2.60
	r^2	0.994							

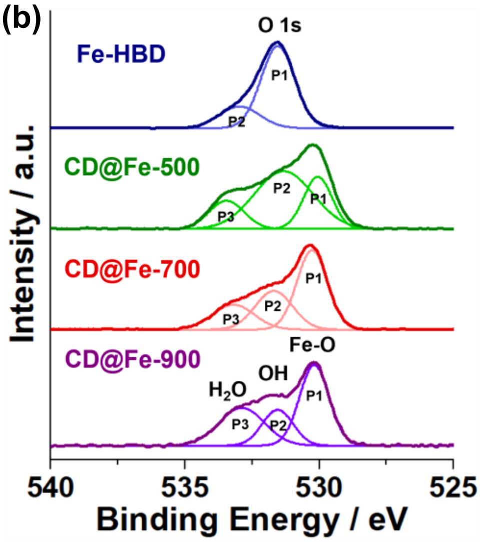

	Peak	P1	P2	P3
Fe-HBD	Position (eV)	531.54	532.97	
	FWHM	1.48	1.77	
	Area (%)	76.0	24.0	
	r^2	0.999		
CD@Fe-500	Position (eV)	530.05	531.32	533.44
	FWHM	1.24	1.52	2.57
	Area (%)	25.2	58.0	16.8
	r^2	0.999		
CD@Fe-700	Position (eV)	530.27	531.68	533.19
	FWHM	1.34	1.64	1.83
	Area (%)	49.3	29.5	21.2
	r^2	0.999		
CD@Fe-900	Position (eV)	530.18	531.54	532.88
	FWHM	1.30	1.35	1.99
	Area (%)	46.0	21.1	32.9
	r^2	0.998		

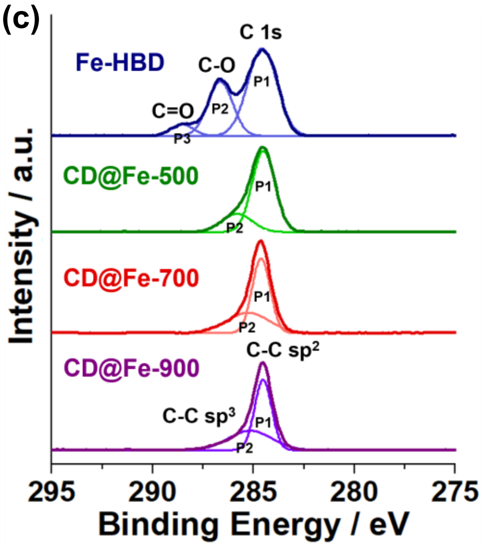

	Peak	P1	P2	P3
Fe-HBD	Position (eV)	284.57	286.65	288.53
	FWHM	1.71	1.44	1.30
	Area (%)	61.6	32.2	6.17
	r^2	0.998		
CD@Fe-500	Position (eV)	284.52	285.82	
	FWHM	1.33	1.75	
	Area (%)	77.2	22.8	
	r^2	0.999		
CD@Fe-700	Position (eV)	284.63	285.27	
	FWHM	1.06	2.48	
	Area (%)	61.0	39.0	
	r^2	0.999		
CD@Fe-900	Position (eV)	284.57	285.14	
	FWHM	1.02	2.80	
	Area (%)	61.1	38.9	
	r^2	0.999		

Figure S6. NO_3RR performance of CD@Fe-500, CD@Fe-700, and CD@Fe-900 under neutral conditions (0.25 M Na_2SO_4) with/without 0.5 M $NaNO_3$: (a) LSV curves, (b) NH_3 yield rates, (c) FEs, and (d-f) CA curves.

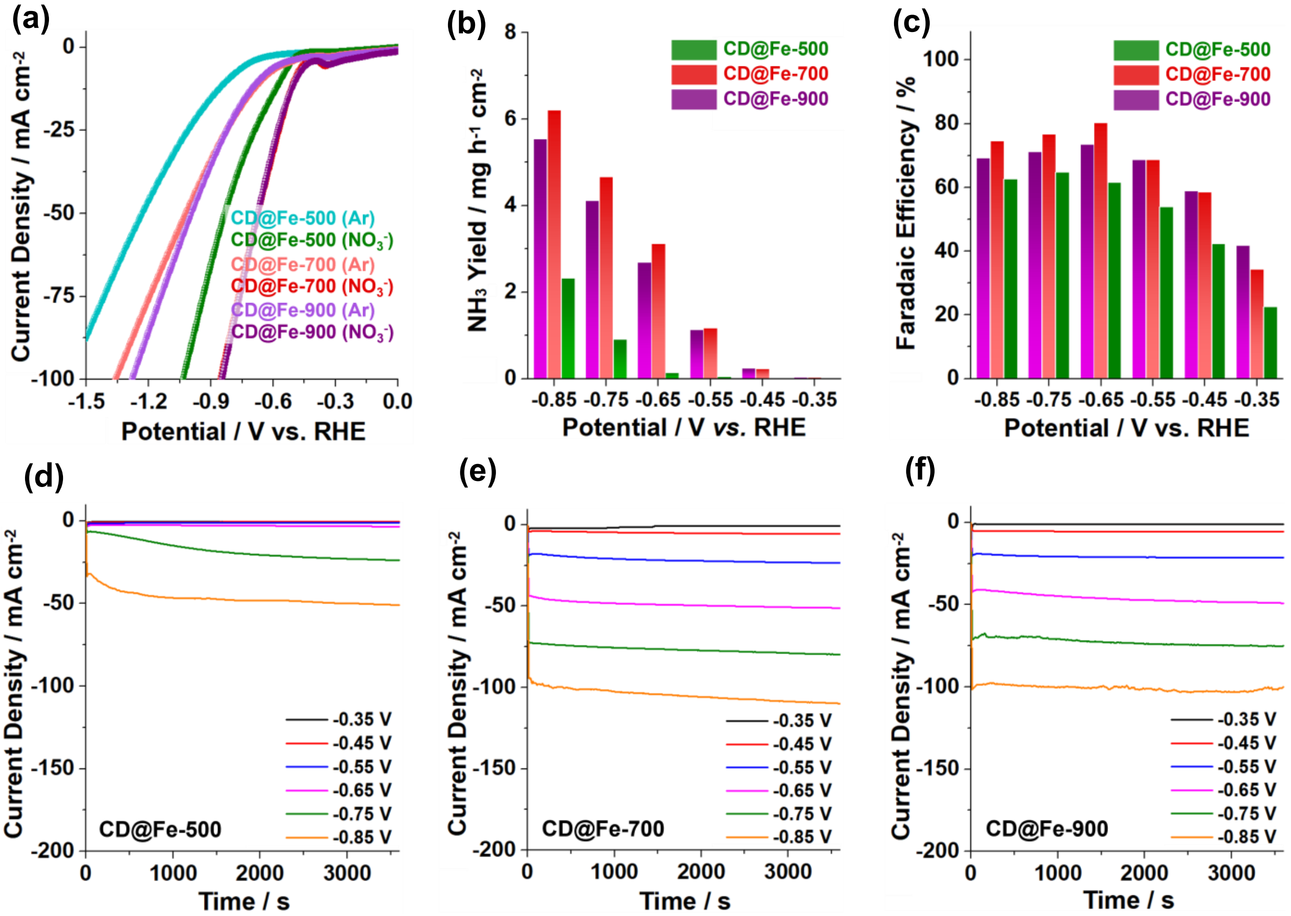

Figure S7. CA curves of the NO_3RR process catalyzed by (a) CD@Fe-500, (b) CD@Fe-700, and (c) CD@Fe-900 under basic conditions (1 M KOH with 0.1 M $NaNO_3$).

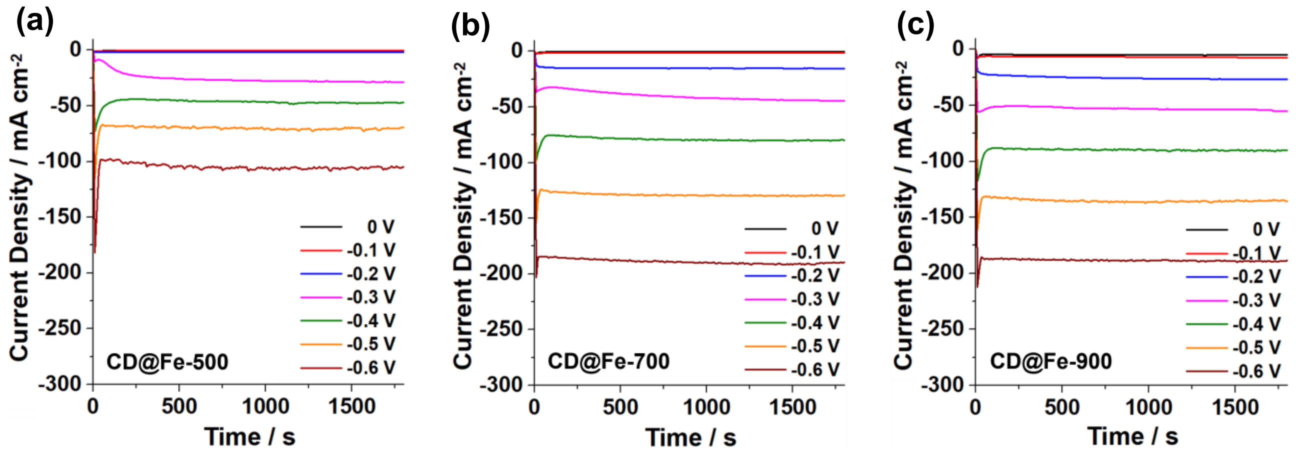

Figure S8. (a-b) UV/vis absorption spectra of a colorimetric indophenol blue-treated ammonia solution and calibration curves under basic conditions (1 M KOH with 0.1 M $NaNO_3$). UV/vis absorption spectra of a colorimetric indophenol blue-treated ammonia solutions obtained through the NO_3RR process catalyzed by (c) CD@Fe-500, (d) CD@Fe-700, and (e) CD@Fe-900 electrocatalysts.

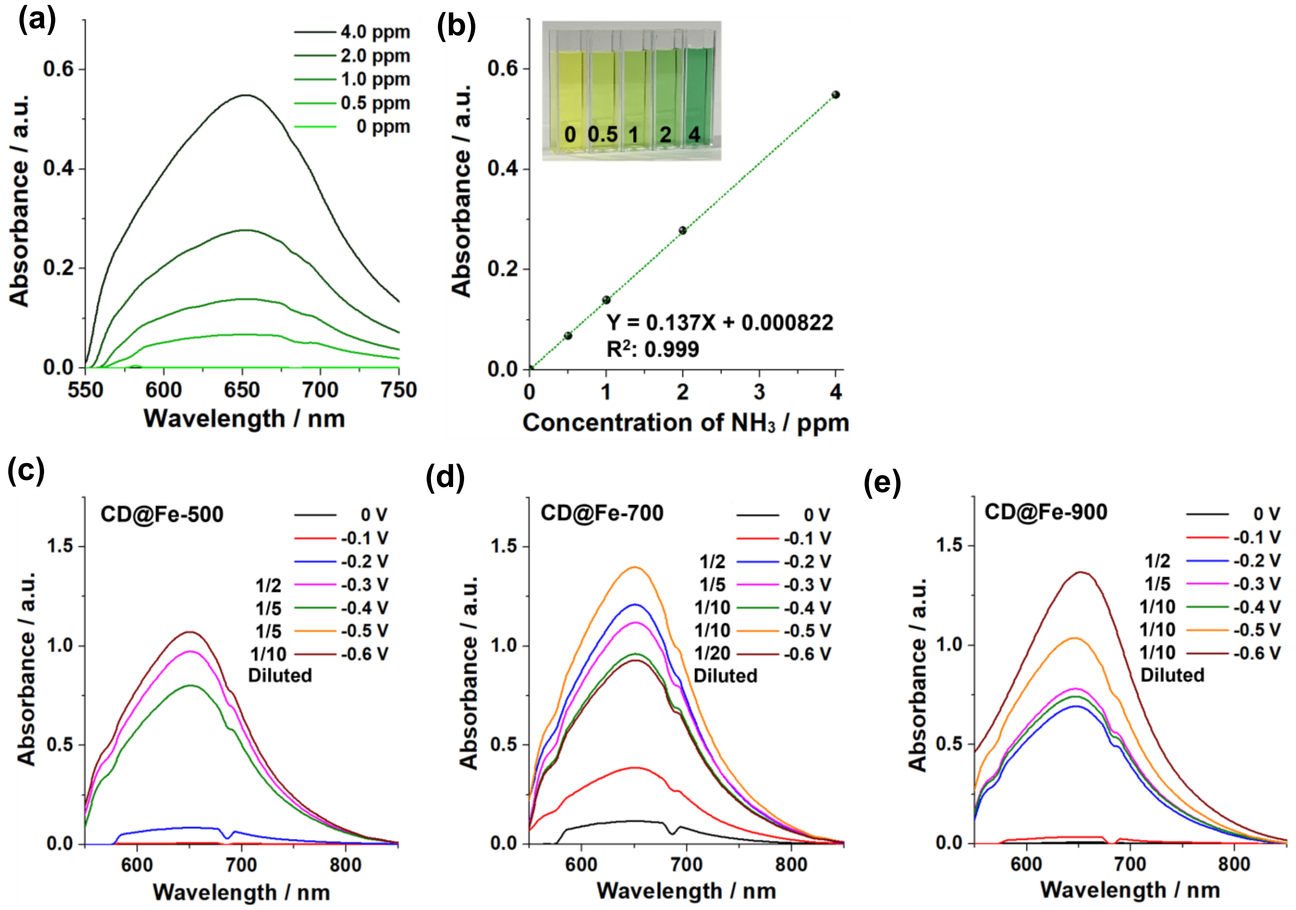

Figure S9. (a) A photograph of the in-situ Raman setup used to investigate intermediates generated during the NO_3RR. (b) In-situ Raman spectra of the NO_3RR process catalyzed by CD@Fe-700 under applied potentials from 0.2 to -0.5 V *vs.* RHE (1 M KOH with 0.1 M $NaNO_3$).

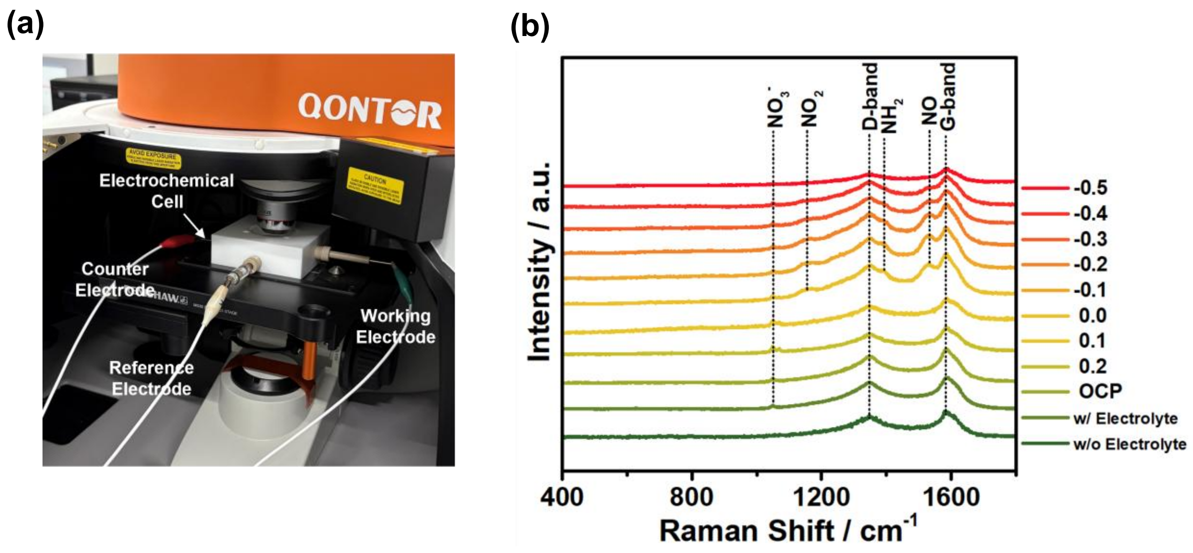

Figure S10. Scan rate-dependent cyclic voltammograms of (a) CD@Fe-500, (b) CD@Fe-700, and (c) CD@Fe-900 under basic conditions (1 M KOH).

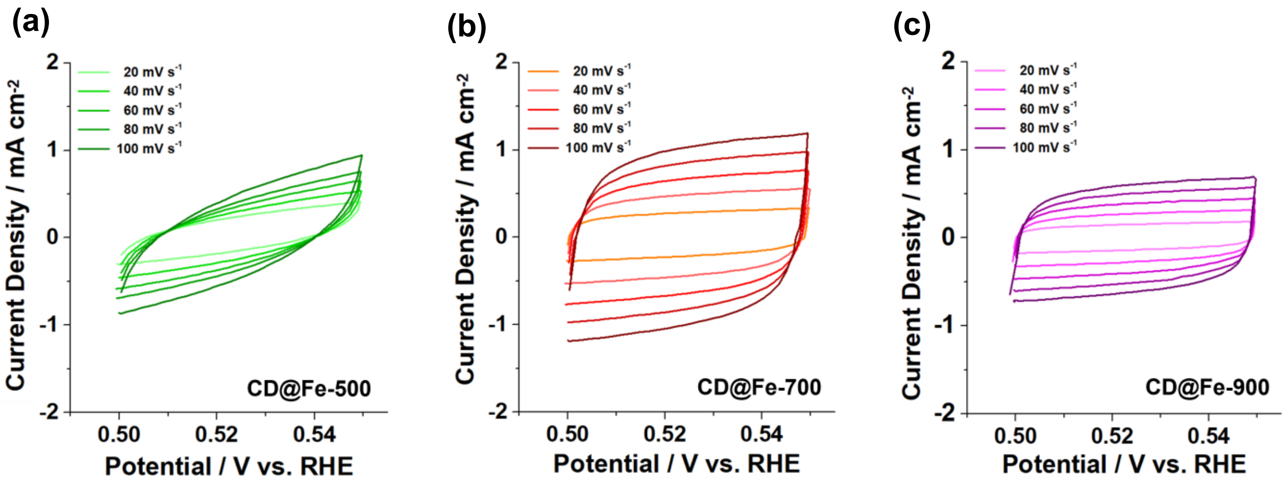

Figure S11. Diffusion coefficients of NO_3RR process catalyzed by CD@Fe-500, CD@Fe-700, and CD@Fe-900 in 1 M KOH with 0.1 M $NaNO_3$ at -0.2 V vs RHE (The diffusion coefficients were calculated based on the Cottrell equation).

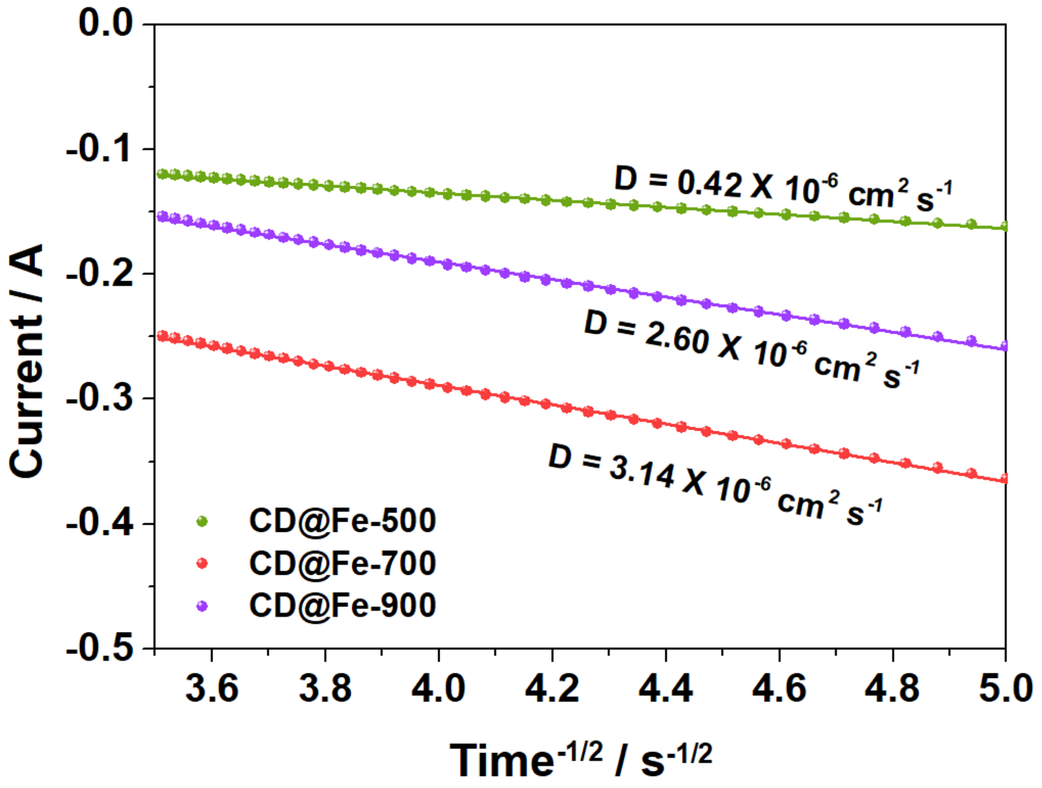

Figure S12. Analysis of the Nyquist plots of CD@Fe-500, CD@Fe-700, and CD@Fe-900.

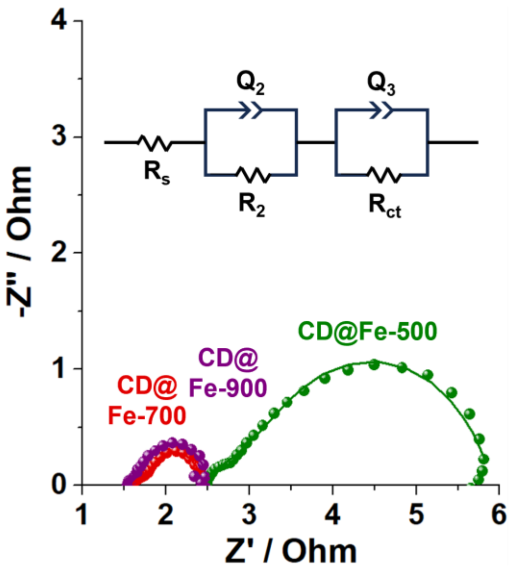

Materials	CD@Fe-500	CD@Fe-700	CD@Fe-900
R_s	2.422	1.597	1.534
Q_2	0.019	0.034	0.055
a_2	0.457	0.619	0.598
R_2	1.052	0.214	0.220
Q_3	4.191e-3	0.045	0.018
a_3	0.835	0.912	0.927
R_{ct}	2.458	0.626	0.756

Figure S13. Nyquist plots of (a) CD@Fe-500, (b) CD@Fe-700, and (c) CD@Fe-900 at various applied potentials (0 to -0.7 V *vs.* RHE) in 1 M KOH with 0.1 M $NaNO_3$. (d) R_{ct} values as a function of applied potential.

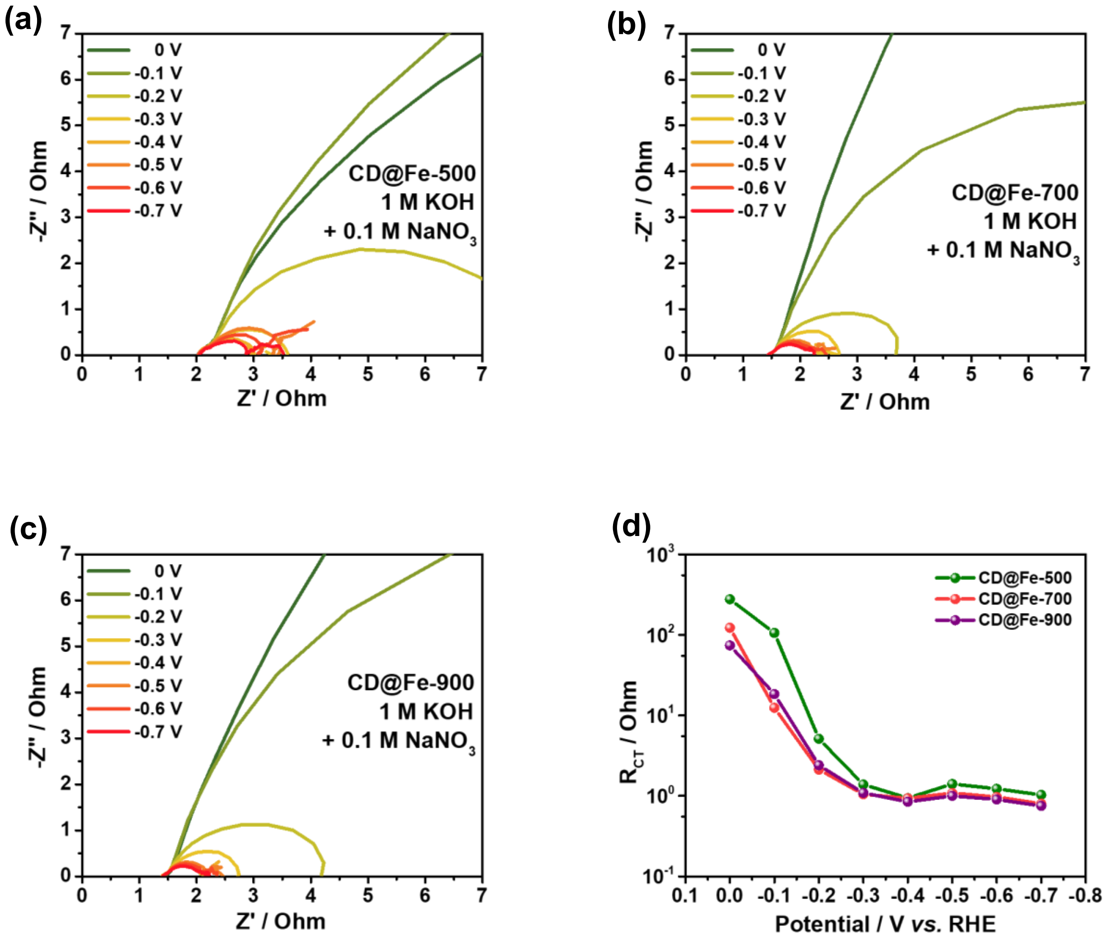

Figure S14. (a) Solution and charge transfer resistances of CD@Fe-700 at various potentials (0 ~ -0.7 V vs RHE) under 1 M KOH and 1 M KOH + 0.1 M NO_3^-. (b) Comparison of R_{ct} between HER and NO_3RR conditions.

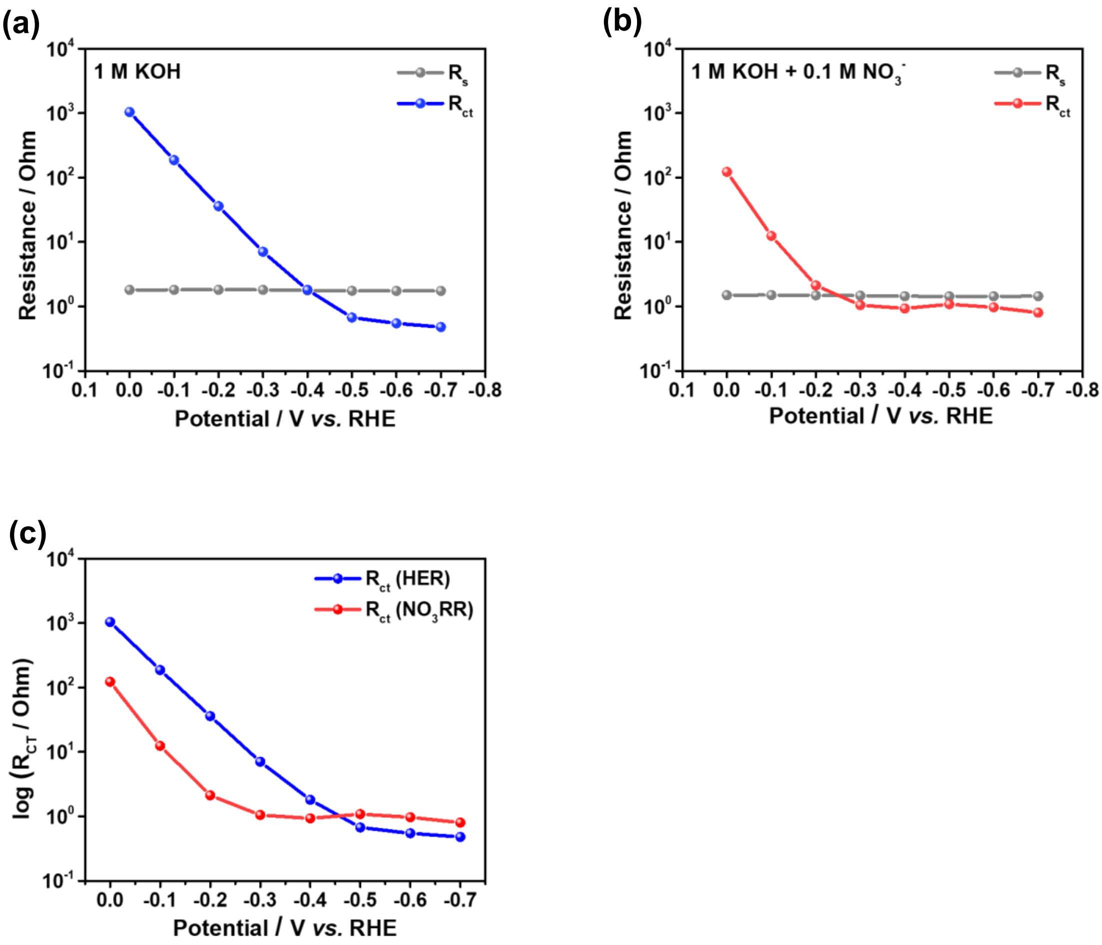

Figure S15. (a) Recyclability performance of the CD@Fe-700 catalyst for the electrochemical NO_3RR to ammonia at -0.3 V (vs. RHE) under basic conditions (1 M KOH with 0.1 M $NaNO_3$). and (b) corresponding UV/vis spectra of generated ammonia solutions.

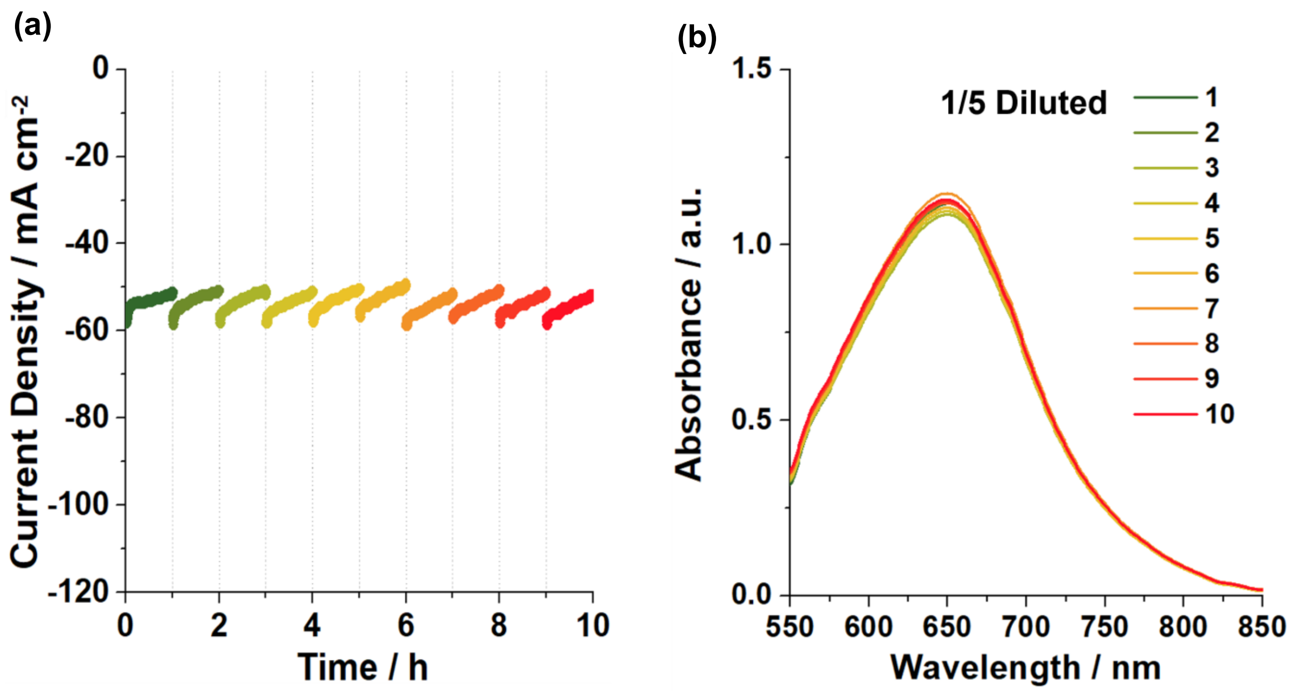

Figure S16. (a) TEM images of CD@Fe-700 before and after electrochemical reactions for 100 h. (b) EDS-based elemental mapping images of CD@Fe-700 recovered after the electrochemical reactions for 100 h.

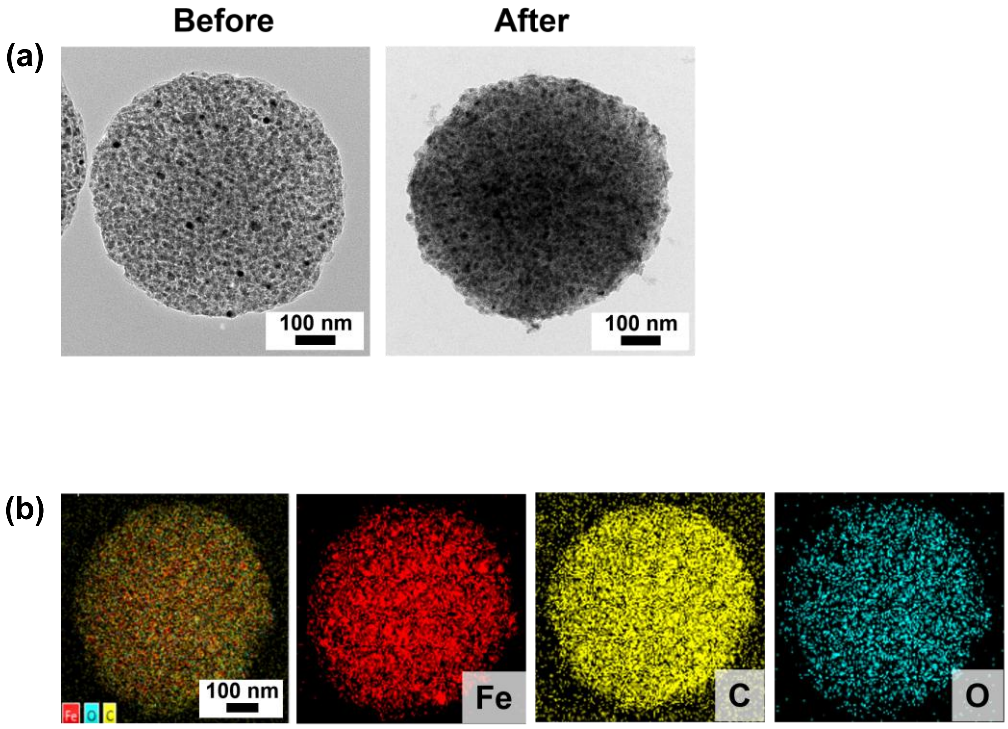

Figure S17. XPS (a) Fe 2p orbitals and (b) O 1s orbital peaks of CD@Fe-700 before use for the fabrication of electrodes, after contact with basic electrolyte (1 M KOH + 0.1 M $NaNO_3$), and after electrochemical reactions for 100 h.

(a)

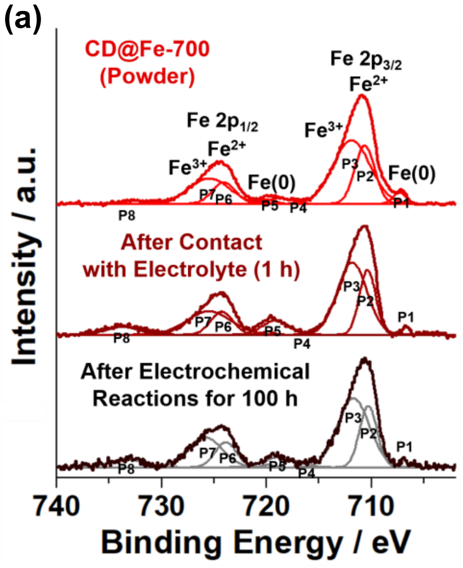

CD@Fe-700 (Powder)	Peak	P1	P2	P3	P4	P5	P6	P7	P8
	Position (eV)	707.28	710.64	711.89	718.73	720.05	724.06	725.46	732.34
	FWHM	1.45	2.23	4.16	2.68	1.67	2.56	4.52	3.18
	Area (%)	2.61	21.2	43.3	2.46	1.17	8.99	18.8	1.53
	r^2	0.997							
CD@Fe-700 (contact)	Peak	P1	P2	P3	P4	P5	P6	P7	P8
	Position (eV)	706.71	710.37	711.83	719.13	719.85	724.25	725.49	733.72
	FWHM	0.764	1.82	3.33	2.92	1.22	2.47	4.90	3.49
	Area (%)	1.08	19.3	39.5	6.83	0.630	9.29	18.9	4.5
	r^2	0.993							
CD@Fe-700 (100 h)	Peak	P1	P2	P3	P4	P5	P6	P7	P8
	Position (eV)	706.89	710.3	711.71	718.67	719.89	723.87	725.86	733.37
	FWHM	0.763	1.95	3.58	3.55	1.15	2.35	3.89	3.61
	Area (%)	0.985	19.4	40.0	5.84	0.473	9.57	18.9	4.90
	r^2	0.990							

(b)

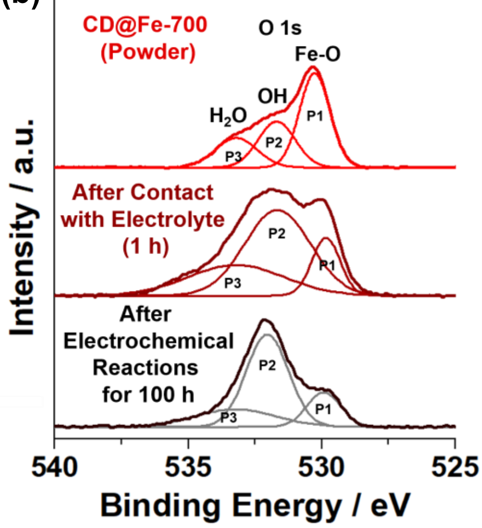

CD@Fe-700 (Powder)	Peak	P1	P2	P3
	Position (eV)	530.27	531.68	533.19
	FWHM	1.34	1.64	1.83
	Area (%)	49.3	29.5	21.2
	r^2	0.999		
CD@Fe-700 (contact)	Peak	P1	P2	P3
	Position (eV)	529.84	531.65	533.20
	FWHM	1.24	2.90	4.25
	Area (%)	15.9	55.2	28.9
	r^2	0.998		
CD@Fe-700 (100 h)	Peak	P1	P2	P3
	Position (eV)	529.92	532.04	533.22
	FWHM	1.49	1.86	3.49
	Area (%)	18.0	60.4	21.6
	r^2	0.998		

Figure S18. (a) LSV curves and (b) Nyquist plots at OCV of NO_3RR process catalyzed by CD@Fe-700 in H-type and zero-gap cells. Polarization curves under (c) H-type cell and (d) zero-gap cell configurations at a scan rate of 5 mV/s using 1 M KOH with 0.1 M $NaNO_3$ as the catholyte and 1 M KOH as the anolyte.

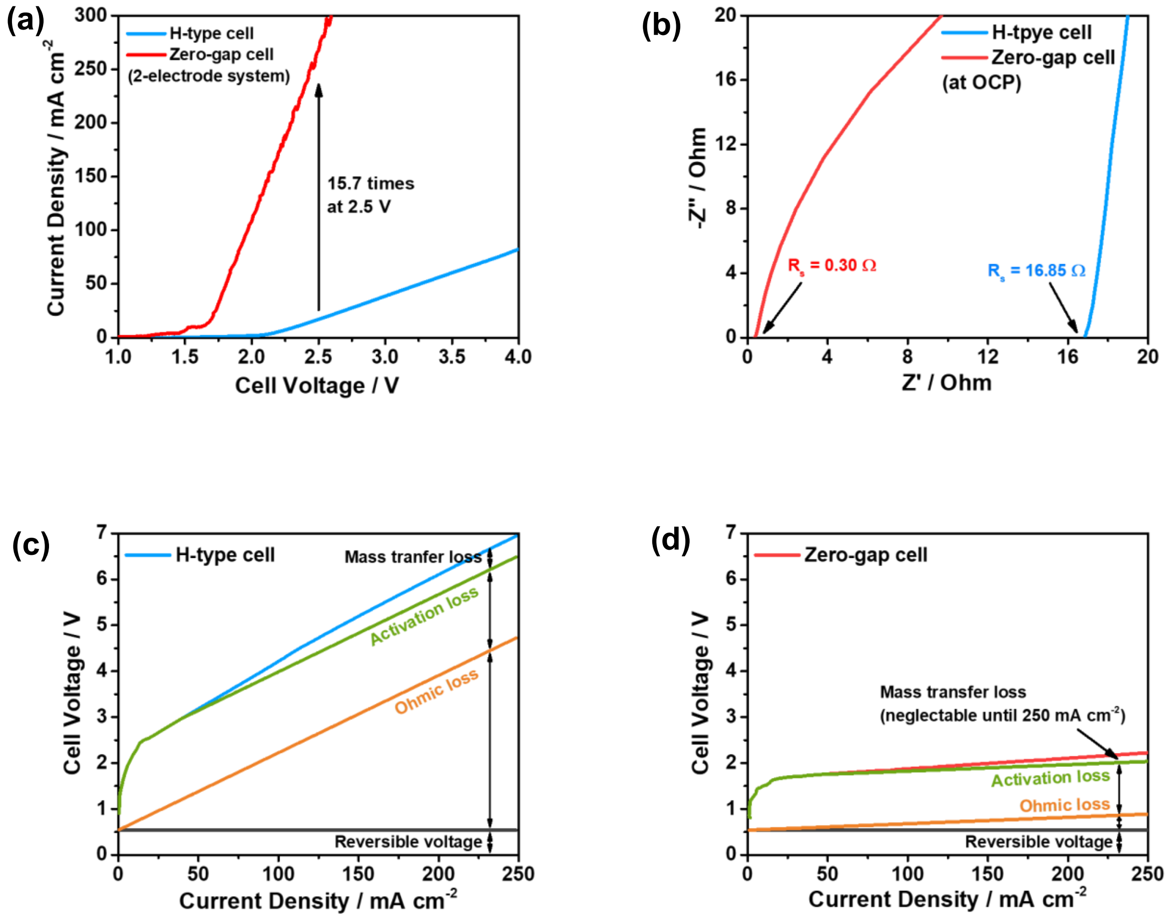

Figure S19. (a) Cell voltage-dependent CA curves and (b) corresponding UV/vis spectra of generated ammonia solutions by zero-gap cells bearing CD@Fe-700 under basic conditions (1 M KOH with 0.1 M $NaNO_3$).

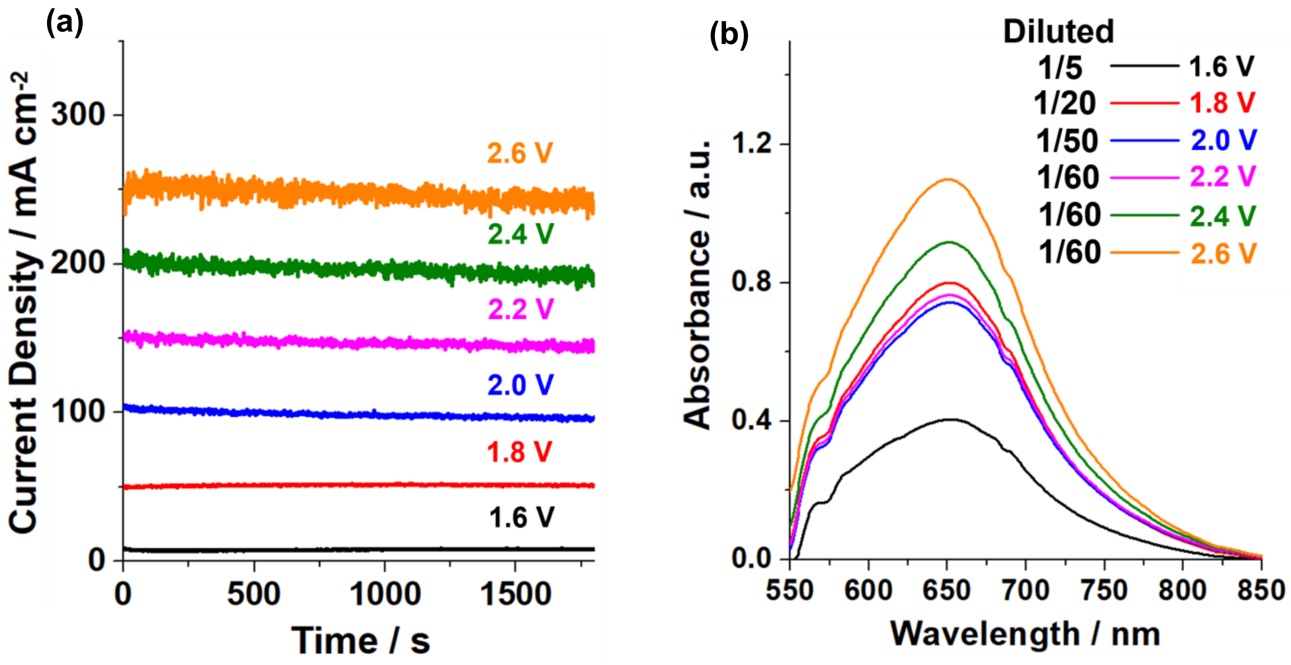

Figure S20. Top view (left) and side view (right) of the slab models for (a) Fe_3O_4 (311) and (b) α-Fe (110) surfaces. The considered adsorption sites for adsorbates were marked by the black circles.

(a)

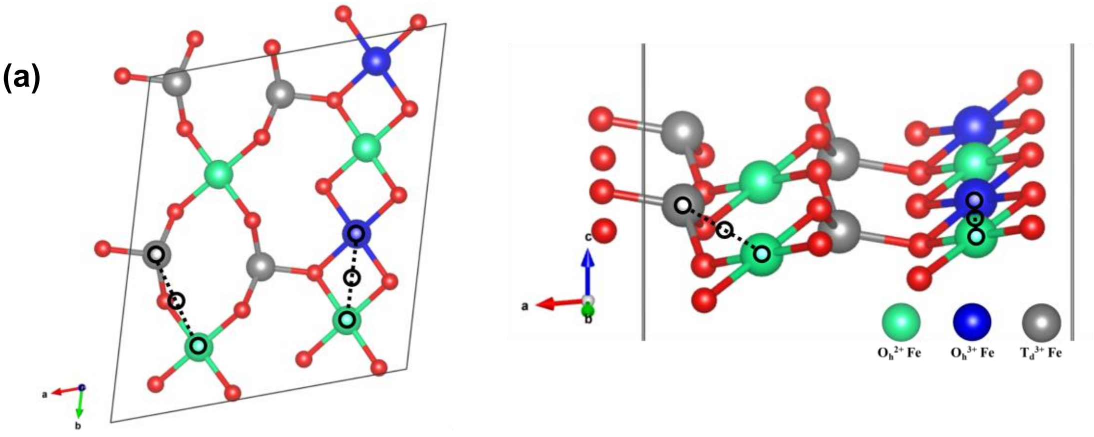

(b)

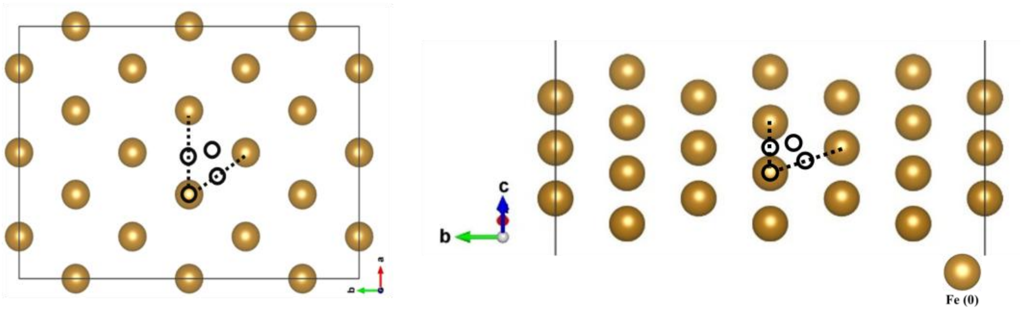

Figure S21. Top and side views for the optimized geometries and the calculated adsorption energies (E^{*}_{NO3} in *eV*) of NO_3 adsorbed on Fe_3O_4 (311) surface in the energy profile. The most stable binding structure was marked by the red box.

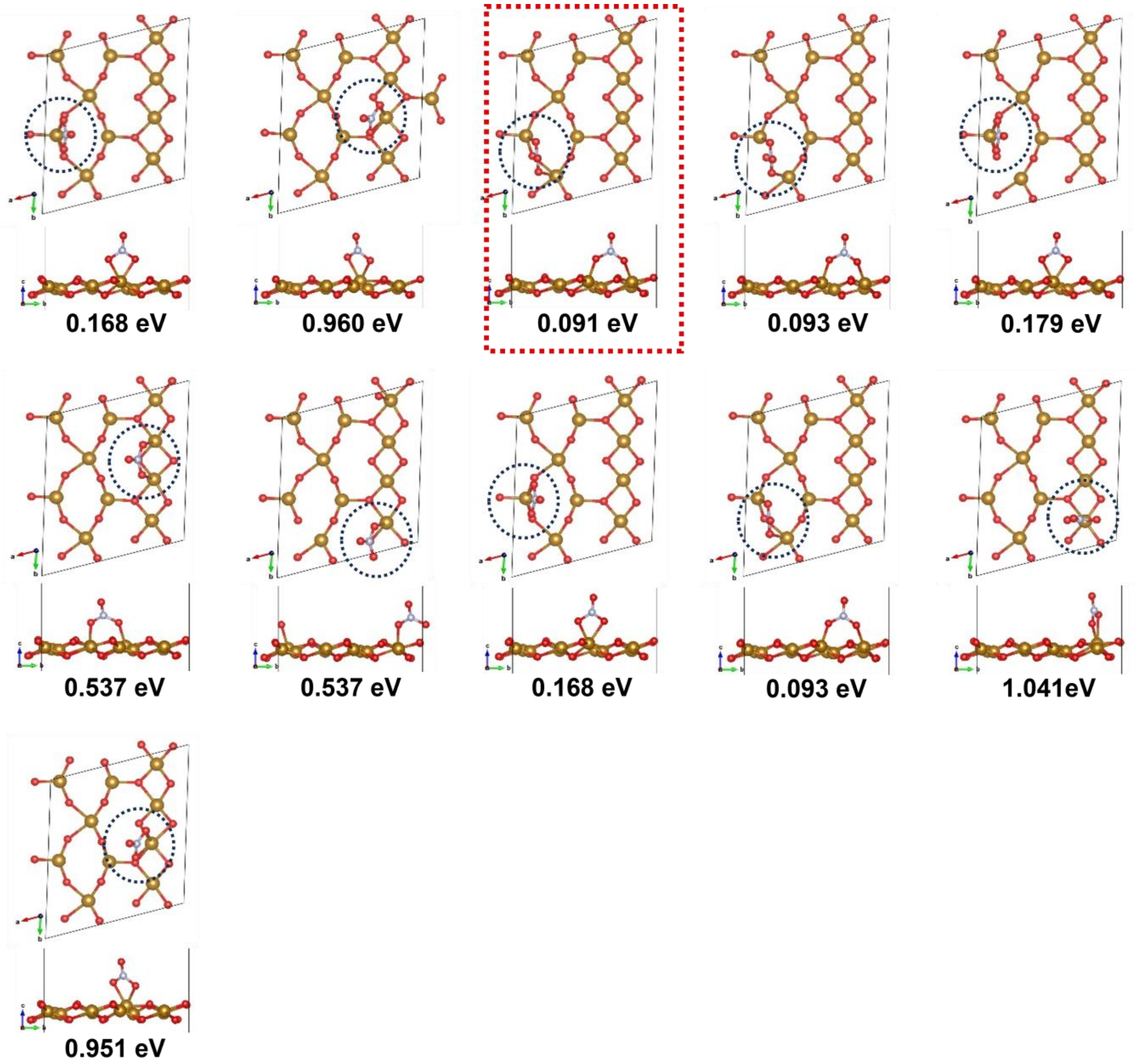

Figure S22. Top and side views for the optimized geometries and the calculated adsorption energies (E^*_{NO2} in *eV*) of NO_2 adsorbed on Fe_3O_4 (311) surface in the energy profile. The most stable binding structure was marked by the red box.

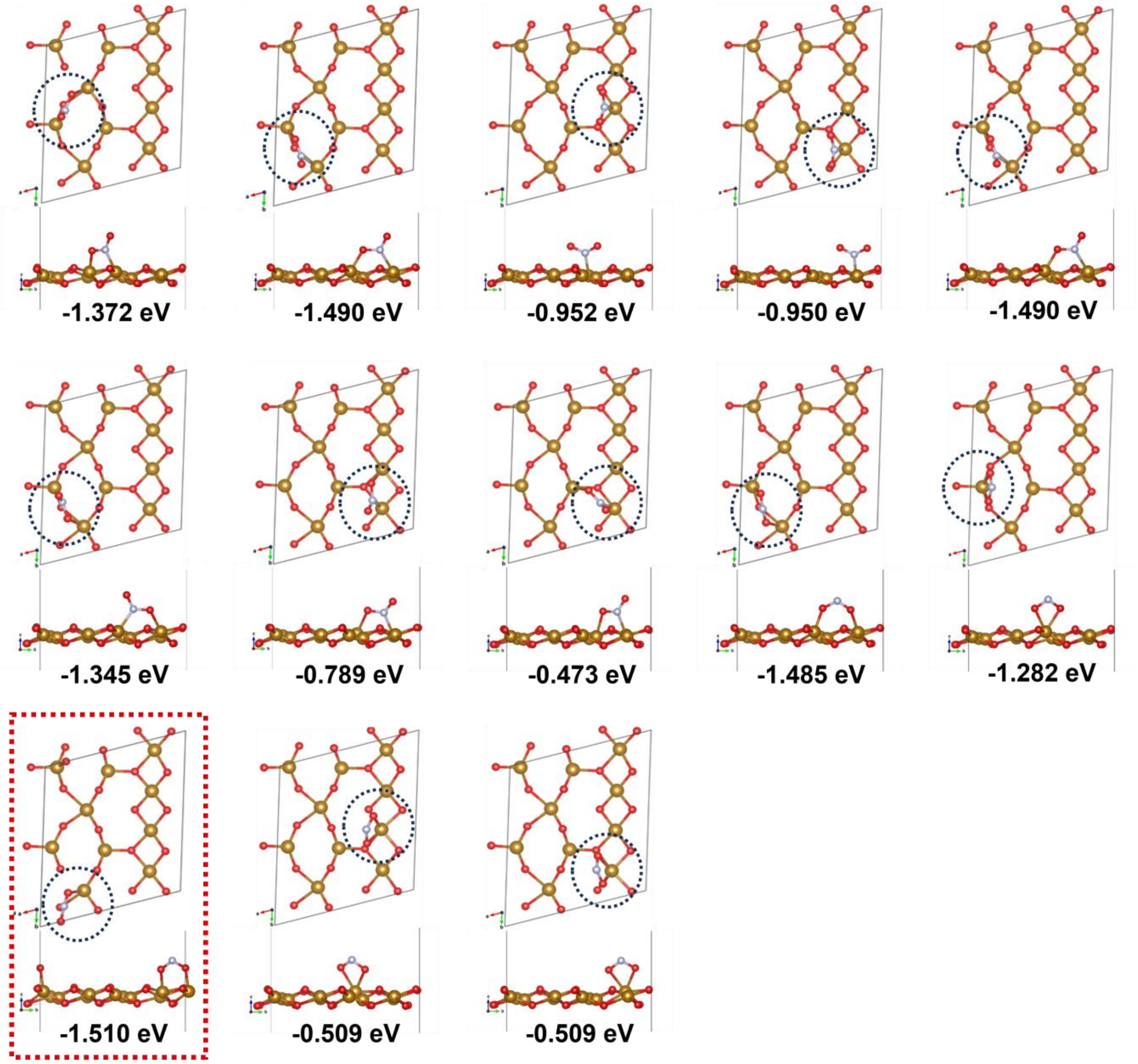

Figure S23. Top view (top) and side view (bottom) of the initally designed and finally optimized structures for the intermediates, namely *HNO, *NO + *H, *HNOH, and *HNO + *H on Fe_3O_4 (311) surface.

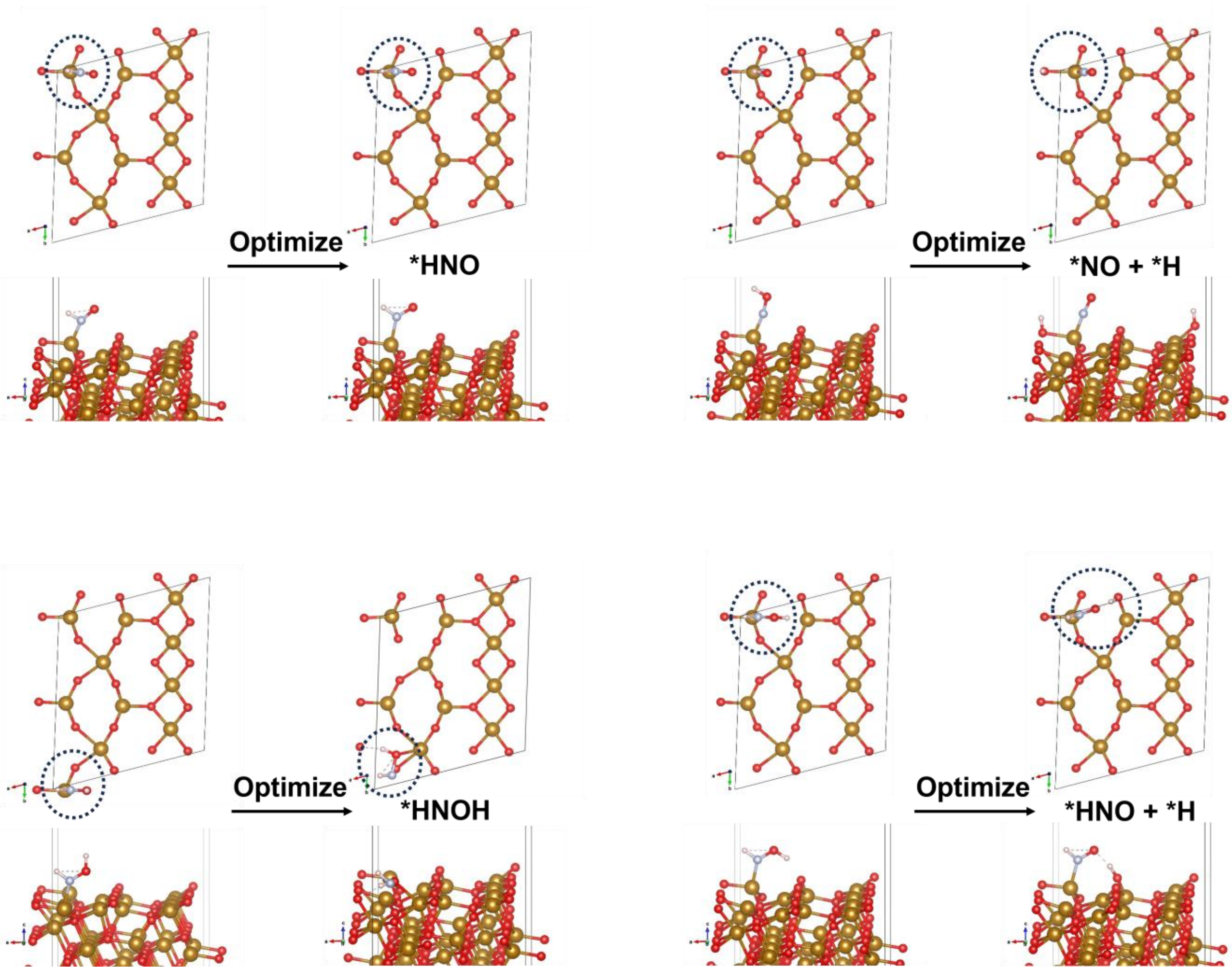

Figure S24. Top and side views for the optimized geometries and the calculated adsorption energies (E^{*}_{NO3} in *eV*) of NO_3 adsorbed on α-Fe (110) surface in the energy profile. The most stable binding structure was marked by the green box.

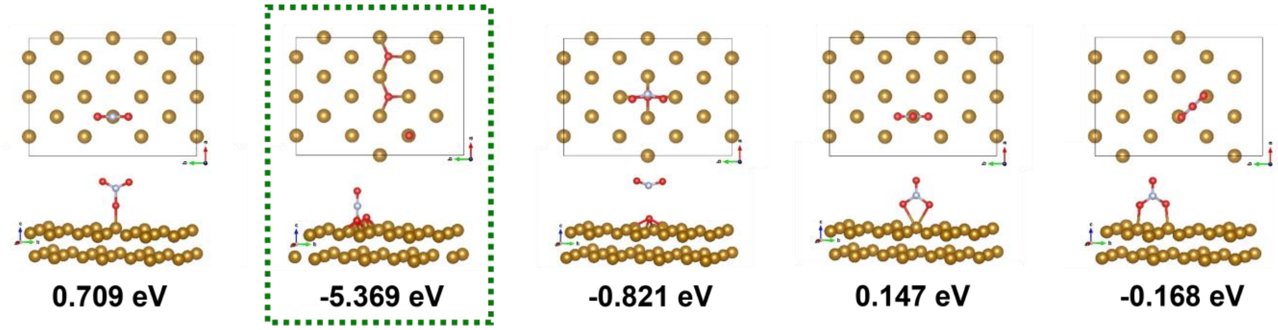

Figure S25. Top and side views for the optimized geometries and the calculated adsorption energies (E^{*}_{NO2} in *eV*) of NO_2 adsorbed on α-Fe (110) surface in the energy profile. The most stable binding structure was marked by the green box.

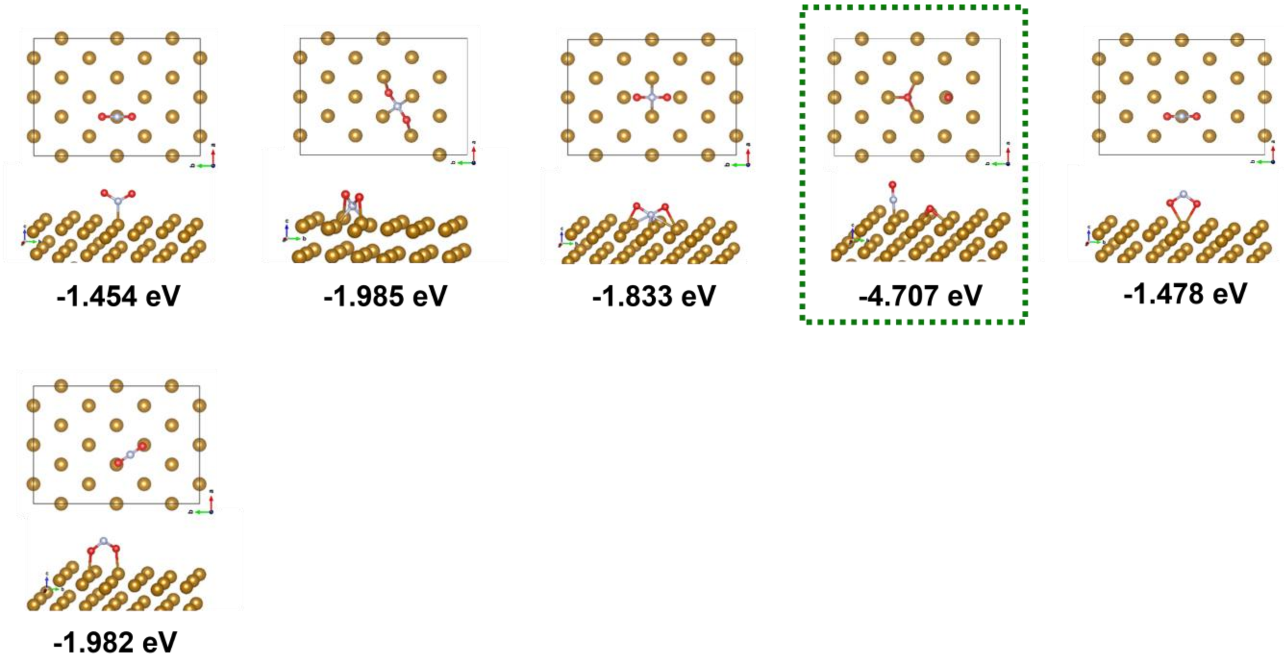

Figure S26. Top view (top) and side view (bottom) of the initally designed and finally optimized structures for the intermediates, namely *NO + *O + *O and *NH + *O on α-Fe (110) surface.

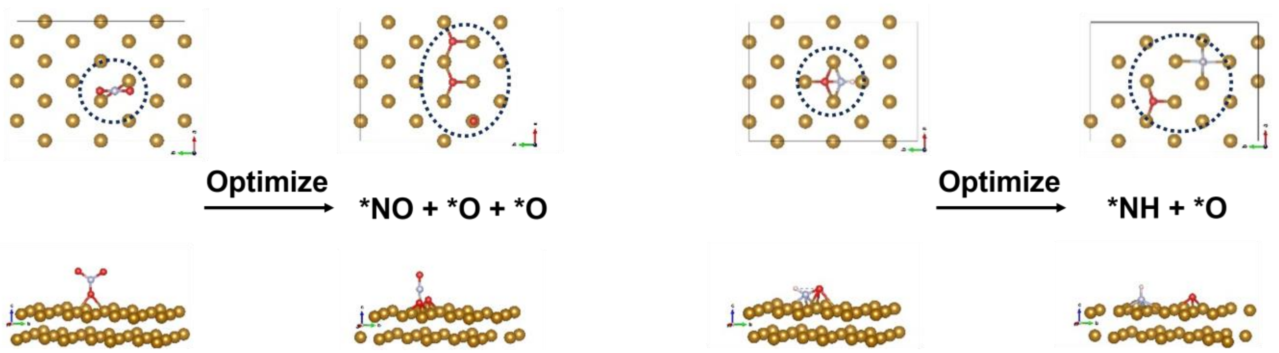

Table S1. Electrocatalytic NO_3RR performance of Fe-based nanocatalysts in the literature.

Entry	Materials	NH_3 yield rate		Potential	FE	EE	N Source	Electrolyte	Year	Ref
		(mg h^{-1} $mg_{cat.}^{-1}$)	(mg h^{-1} cm^{-2})	(V *vs.* RHE)	(%)	(%)				
1	Fe-N-C	5.2	2.1	-0.66	75.0	17.9	0.5 M KNO_3	0.1 M K_2SO_4	2021	[S1]
2	Fe-CNS	-	-	-0.57	78.0	19.5	100 mg L^{-1} NO_3^-	0.02 M Na_2SO_4	2021	[S2]
3	Fe-Ppy-SACs	482.8	2.3	-0.6	100.0	29.5	0.1 M KNO_3	0.1 M KOH	2021	[S3]
4	Fe SACs/g-C_3N_4	9.9	0.9	-0.6	77.3	19.0	50 mg N L^{-1}	0.1 M Na_2SO_4	2022	[S4]
5	FeN_2O_2	46.0	9.2	-0.68	92.0	21.7	0.5 M KNO_3	0.1 M K_2SO_4	2022	[S5]
6	Fe-MoS_2		0.5	-0.5	98.0	25.5	0.1 M KNO_3	0.1 M Na_2SO_4	2022	[S6]
7	FeOOH/CP		2.4	-0.5	92.0	23.9	0.1 M $NaNO_3$	0.1 M PBS	2022	[S7]
8	FeNPs@Mxene		0.5	-0.95	34.0	7.0	100 mg L^{-1} NO_3^-	0.1 M Na_2SO_4	2022	[S8]
9	Co-Fe@Fe_2O_3		0.9	-0.75	85.2	19.4	500 ppm NO_3^-	0.1 M Na_2SO_4	2022	[S9]
10	Fe-Co_3O_4	0.6	0.6	-0.7	95.5	22.3	50 mM KNO_3	0.1 M PBS	2022	[S10]
11	Fe_1/NC-900	6.8	6.8	-0.7	86.7	20.2	0.5 M KNO_3	0.1 M K_2SO_4	2023	[S11]
12	Fe-V_2O_5		12.5	-0.7	98.0	27.4	0.1 M KNO_3	1.0 M KOH	2023	[S12]
13	FeSA/MXene		0.1	-0.79	82.9	18.5	50 mg N L^{-1}	0.1 M Na_2SO_4	2023	[S13]
14	Mo_2CTx:Fe	0.1	0.03	-0.6	70.0	17.2	0.1 M NO_3^-	0.5 M Na_2SO_4	2023	[S14]
15	Fe-CoS_2		10.8	-0.6	97.5	24.0	0.1 M $NaNO_3$	0.5 M Na_2SO_4	2023	[S15]
16	Fe_2TiO_5	0.7	0.1	-1.0	87.6	17.7	0.1 M $NaNO_3$	PBS	2023	[S16]
17	Fe_1/Cu_2-Co_3O_4		3.0	-0.79	98.5	22.0	100 ppm NO_3^-	0.2 M Na_2SO_4	2023	[S17]
18	Cu-$LaFeO_3$		0.8	-1.0	71.9	14.5	0.1 M $NaNO_3$	0.1 M Na_2SO_4	2023	[S18]
19	Cu/Fe-TiO_2		0.8	-0.7	91.2	20.8	50 ppm $NaNO_3$	0.5 Na_2SO_4	2023	[S19]
20	Fe/Cu-HNG		1.3	-0.3	92.5	32.7	0.1 M KNO_3	1 M KOH	2023	[S20]
21	Fe_2P		2.1	-0.55	96.0	29.1	0.2 M $NaNO_3$	0.5 M NaOH	2023	[S21]
22	Fe@Cu_1FeOx		2.0	-0.64	95.4	22.9	1000 mg L^{-1} NO_3^-	0.1 M K_2SO_4	2024	[S22]
23	Fe SAC/NC	22.5	4.5	-0.8	98.2	26.1	1000 ppm KNO_3	1 M KOH	2024	[S23]
24	FeNP/FeSA-N-C	4.1	14.6	-0.9	86.2	18.2	0.1 M KNO_3	0.5 M K_2SO_4	2024	[S24]
25	3×Fe_2O_3/Fe-N-C		23.8	-0.4	95.0	31.5	0.16 M KNO_3	1 M KOH	2024	[S25]
26	Cu-Fe-N-C		20.7	-0.8	95.1	25.3	0.1 M KNO_3	1 M KOH	2024	[S26]
27	Fe_3C NPs@NCF		8.4	-0.8	96.9	25.8	0.1 M NO_3^-	0.1 M NaOH	2024	[S27]
28	$Co_1Fe_{1.5}$/C	48.2	6.0	-1.1	90.8	17.5	0.1 M KNO_3	1 M K_2SO_4	2024	[S28]
29	FePc@CNT	5.95	2.4	-0.45	94.0	30.2	0.1 M $NaNO_3$	1 M NaOH	2024	[S29]
30	$FeNi_3$@P-NC		9.6	-0.7	93.0	26.0	0.1 M NO_3^-	0.1 M KOH	2024	[S30]
31	Fe_2O_3-Ni-CB		5.2	-0.5	94.6	29.5	0.5 M $NaNO_3$	0.1 M NaOH	2024	[S31]
32	Fe SAC/FeP@C	14	7.0	-0.8	95.6	17.0	0.5 M KNO_3	0.05 M H_2SO_4	2024	[S32]
33	Fe_2O_3/CC		5.6	-0.9	84.9	17.9	0.1 M $NaNO_3$	0.5 M Na_2SO_4	2024	[S33]
34	Ru-Fe_2O_3		4.7	-0.8	72.9	16.1	0.1 M $NaNO_3$	0.5 M Na_2SO_4	2024	[S34]
35	Fe/ZnO		1.9	-0.7	83.0	23.2	0.05 M $NaNO_3$	0.1 M KOH	2024	[S35]
36	CuFe-P/IF		2.7	-0.2	72.0	27.2	0.1 M $NaNO_3$	1 M KOH	2024	[S36]
37	FeP–PNC-M	14.7	4.4	-0.9	85.4	18.0	0.1 M KNO_3	0.1 M K_2SO_4	2025	[S37]
38	Fe_3C@NG-10	8.1	1.6	-0.5	94.0	24.5	25 mM $NaNO_3$	0.5 M Na_2SO_4	2025	[S38]
39	Fe_2N@NC	10.5	2.1	-0.5	96.1	25.0	0.025 M NO_3^-	0.5 M Na_2SO_4	2025	[S39]
40	Co-$SrFeO_3$	16.1	0.1	-0.9	81.5	17.2	0.1 M NaNO3	0.1 M PBS	2025	[S40]
41	MFe_2O_4		3.4	-0.8	95.2	21.1	0.1 M KNO_3	0.1 M K_2SO_4	2025	[S41]
42	$Co_{0.1}$@MIL-Fe		1.4	-0.9	98.0	20.7	50 mg L^{-1} $NaNO_3$	0.1 M Na_2SO_4	2025	[S42]
43	Fe_1Mn_2/NOPC		0.9	-0.8	87.7	19.4	0.1 M KNO_3	0.1 M K_2SO_4	2025	[S43]
44	FeCoNiMgZnO		1.9	-0.6	96.1	23.6	0.05 M $NaNO_3$	0.5 M Na_2SO_4	2025	[S44]
45	Co_4Fe_6		2.7	-0.69	98.6	23.1	500 ppm NO_3^-	0.1 M Na_2SO_4	2025	[S45]
46	Fe-N_4/CNCl		0.8	-0.6	93.5	23.0	0.5 M KNO_3	0.1 M Na_2SO_4	2025	[S46]
47	γ-Fe_2O_3/BCN	4.9	4.9	-0.59	95.0	23.5	0.1 M $NaNO_3$	0.1 M PBS	2025	[S47]
48	FeCu-NPCS	~38	~3.8	-0.6	97.4	28.7	0.1 M KNO_3	1 M KOH	2025	[S48]
49	$Co_1Ni_2Cu_1Mn_1Fe_1$		3.3	-0.6	92.0	27.1	0.05 M NO_3^-	0.1 M KOH	2025	[S49]
50	Ag-GO-Hemin-His		3.8	-0.5	91.9	28.7	0.1 M NO_3^-	1 M KOH	2025	[S50]
51	Mo_1Fe_1Pd	47.6	4.8	-0.7	94.6	26.5	1 M KNO_3	1 M KOH	2025	[S51]
52	CD@Fe-700		4.1	**-0.3**	**92.3**	**32.6**	0.1 M $NaNO_3$	1 M KOH	This Work	
53	CD@Fe-700		1.8	**-0.2**	**92.0**	**34.7**	0.1 M $NaNO_3$	1 M KOH	This Work	

[S1] Z. -Y. Wu, M. Karamad, X. Yong, Q. Huang, D. A. Cullen, P. Zhu, C. Xia, Q. Xiao, M. Shakouri, F. -Y. Chen, J. Y. Kim, Y. Xia, K. Heck, Y. Hu, M. S. Wong, Q. Li, I. Gates, S. Siahrostami, H. Wang, *Nat. Commun.* **2021**, *12*, 2870.

[S2] J. Li, M. Li, N. An, S. Zhang, Q. Song, Y. Yang, X. Liu, *Proc. Natl. Acad. Sci. U. S. A.* **2021**, *118*, e2105628118.

[S3] P. Li, Z. Jin, Z. Fang, G. Yu, *Energy Environ Sci.* **2021**, *14*, 3522-3531.

[S4] Q. Song, M. Li, X. Hou, J. Li, Z. Dong, S. Zhang, L. Yang, *Appl. Catal. B: Envrion.* **2022**, *317*, 121721.

[S5] W. -D. Zhang, H. Dong, L. Zhou, H. Xu, H. -R. Wang, X. Yan, Y. Jiang, J. Zhang, Z. -G. Gu, *Appl. Catal. B: Environ.* **2022**, 317, 121750.

[S6] J. Li, Y. Zhang, L. Zheng, E. Petit, K. Qi, Y. Zhang, H. Wu, W. Wang, A. Tiberj, X. Wang, M. Chhowalla, L. Lajaunie, R. Yu, D. Voiry, *Adv. Funct. Mater.* **2022**, *32*, 2108316.
[S7] Q. Liu. Q. Liu, L. Xie, Y. Ji, T. Li, B. Zhang, N. Li, B. Tang, Y. Liu, S. Gao, Y. Luo, L. Yu, Q. Kong, X. Sun, *ACS Appl. Mater. Interfaces* **2022**, *14*, 17312-17318.
[S8] W. -J. Sun, L. -X. Li, H. -Y. Zhang, J. -H. He, J. -M. Lu, *ACS Sustainable Chem. Eng.* **2022**, *10*, 5958-5965.
[S9] S. Zhang, M. Li, J. Li, Q. Song, X. Liu, *Proc. Natl. Acad. Sci. U. S. A.* **2022**, *119*, e21155504119.
[S10] P. Wei, J. Liang, Q. Liu, L. Xie, X. Tong, Y. Ren, T. Li, Y. Luo, N. Li, B. Tang, A. M. Asiri, M. S. Hamdy, Q. Kong, Z. Wang, X. Sun, *J. Colloid. Interface Sci.* **2022**, *615*, 636-642.
[S11] L. Liu, T. Xiao, H. Fu, Z. Chen, X. Qu, S. Zheng, *Appl. Catal. B: Environ.* **2023**, *323*, 122181.
[S12] N. Zhang, G. Zhang, P. Shen, H. Zhang, D. Ma, K. Chu, *Adv. Funct. Mater.* **2023**, *33*, 2211537.
[S13] Y. Ren. F. Tian, L. Jiin, Y. Wang, J. Yang, S. You, Y. Liu, *Environ. Sci. Technol.* **2023**, *57*, 10458-10466.
[S14] D. F. Abbott, Y. -Z. Xu, D. A. Kuznetsov, P. Kumar, C. R. Müller, A. Fedorov, V. Mougel, *Angew. Chem. Int. Ed.* **2023**, *62*, e202313746.
[S15] N. Zhang, G. Wang, G. Zhang, K. Chen, K. Chu, *Chem. Eng. J.* **2023**, *474*, 145861.
[S16] H. Du, H. Guo, K. Wang, X. Du, B. A. Beshiwork, S. Sun, Y. Luo, Q. Liu, T. Li, X. Sun, *Angew. Chem. Int. Ed.* **2023**, *62*, e202215782.
[S17] M. Song, Y. Xing, Y. Li, D. Liu, E. Han, Y. Gao, Z. Yang, X. Yang, Y. He, *Inorg. Chem.* **2023**, *62*, 16641-16651.
[S18] Q. Yin, H. Zhou, Mater. *Today Commun.* **2023**, *35*, 106048.
[S19] X. Yang, R. Wang, S. Wang, C. Song, S. Lu, L. Fang, F. Yin, H. Liu, *Appl. Catal. B: Environ.* **2023**, *325*, 122360.
[S20] S. Zhang, J. Wu, M. Zheng, X. Jin, Z. Shen, Z. Li, Y. Wang, Q. Wang, X. Wang, H. Wei, J. Zhang, P. Wang, S. Zhang, L. Yu, L. Dong, Q. Zhu, H. Zhang, J. Lu, *Nat. Commun.* **2023**, *14*, 3634.
[S21] T. Chouki, M. Machreki, I. A. Rutkowska, B. Rytelewska, P. J. Kulesza, G. Tyuliev, M. Harb, L. M. Azofra, S. Emin, *J. Environ. Chem. Eng.* **2023**, *11*, 109275.
[S22] B. Zhou, L. Yu, W. Zhang, X. Liu, H. Zhang, J. Cheng, Z. Chen, H. Zhang, M. Li, Y. Shi, F. Jia, Y. Huang, L. Zhang, Z. Ai, *Angew. Chem. Int. Ed.* **2024**, *63*, e202406046.
[S23] X. Cheng, W. Shang, Y. Li, J. Hu, J. Guo, D. Cao, N. Zhang, S. Zhang, S. Song, T. Liu, W. Liu, Y. Shi, *Nano Res.* **2024**, *17*, 6826-6832.
[S24] L. Wang, P. Guo, Y. Han, C. Han, H. Sun, R. Huang, X. Liu, M. Huang, Z. Mao, X. Yan, A. Du, X. Wang, *Chem Catal.* **2024**, *4*, 100936.
[S25] E. Murphy, B. Sun, M. Rüscher, Y. Liu, W. Zang, S. Guo, Y. -H. Chen, U. Hejral, Y. Huang, A. Ly, I. V. Zenyuk, X. Pan, J. Timoshenko, B. R. Cuenya, E. D. Spoerke, P. Atanassov, *Adv. Mater.* **2024**, *36*, 2401133.
[S26] X. Zhang, X. Liu, Z. -F. Huang, L. Gan, S. Zhang, R. Jia, M. Ajmal, L. Pan, C. Shi, X. Zhang, G. Yang, J. -J. Zou, *Energy Environ. Sci.* **2024**, *17*, 6717-6727.
[S27] X. Liu, T. Xie, Z. Cai, Z. Li, L. Zhang, X. Fan, D. Zhao, S. Sun, Y. Luo, Q. Liu, X. Sun, *J. Electroanal. Chem.* **2023**, *933*, 117295.
[S28] Y. Liu, X. Zhong, M. Liu, H. Zhao, Z. Wang, R. Ni, Y. Wang, J. Yang, F. Gao, Y. Li, E. Yuan, A. Yuan, W. Shi, F. Yang, *Appl. Catal. B: Environ. Energy* **2024**, *355*, 124205.
[S29] W. Wang, M. Li, M. Liang, Y. Lu, Q. He, F. Chen, *J. Clean. Prod.* **2024**, *472*, 143514.
[S30] M. Zhang, X. Cheng, X. Yao, J. Chu, F. Bai, C. Sun, Y. -Q. Wang, *J. Colloid Interface Sci.* **2025**, *680*, 632-642.
[S31] Q. Ru, P. Bai, X. Kong, L. Xu, *Chem. Eng. Sci.* **2024**, *298*, 120378.
[S32] J. Song, S. -J. Qian, W. Yang, J. Mu, J. Li, Y. Liu, F. Sun, S. Yu, F. Xu, X. Song, D. Deng, Y. -G. Wang, L. Yan, Y. Ding, *Adv. Funct. Mater.* **2024**, *34*, 2409089.
[S33] T. Li, C. Tang, H. Guo, J. Yang, F. Zhang, G. Yang, Y. Zhou, *Chem. Eng. J.* **2024**, *485*, 149560.
[S34] S. Luo, H. Guo, T. Li, H. Wu, F. Zhang, C. Tang, G. Chen, G. Yang, Y. Zhou, *Appl. Catal. B: Environ. Energy* **2024**, *351*, 123967.
[S35] T. S. Bui, Z. Ma, J. A. Yuwono, P. V. Kumar, G. E. P. O'Conell, L. Peng, Y. Yang, M. Lim, R. Daiyan, E. C. Lovell, R. Amal, *Adv. Funct. Mater.* **2024**, *34*, 2408704.
[S36] G. Wang, C. Wang, X. Tian, Q. Li, S. Liu, X. Zhao, G. I. N. Waterhouse, X. Zhao, X. Lv, J. Xu, *Small* **2024**, *20*, 2311439.
[S37] Z. Fan, B. Pang, W. Chen, F. Cui, X. Qi, X. Wu, N. Wang, W. Yu, G. He, *Chem. Eng. J.* **2025**, *507*, 160088.
[S38] T. Rao, J. Zhan, Y. Du, L. H. Zhang, F. Yu, *ChemSusChem* **2025**, *18*, e202402460.
[S39] Y. Chen, T. Rao, J. Zhan, L. H. Zhang, F. Yu, *Chem. Commun.* **2025**, *61*, 7684–7687.
[S40] W. M. Syoum, L. Niu, K. Zhang, H. Guo, J. S. Chen, T. Li, *ChemNanoMat* **2025**, *11*, e202400632.
[S41] J. Ding, L. Zhang, Z. Wei, Z. Wang, Q. Liu, G. Hu, J. Luo, X. Liu, *Small* **2025**, *21*, 2411317.
[S42] X. Wang, D. Wang, H. Ma, G. Wang, *J. Colloid Interface Sci.* **2025**, *677*, 369–377.
[S43] Y. Wang, L. Zhang, C. Wang, Z. Wang, Y. Feng, X. Liu, *Chem. Commun.* **2025**, *61*, 4399–4402.
[S44] C. Xu, H. Mou, D. Li, D. Zhang, L. Feng, S. Lv, C. Song, S. Sun, J. Song, D. Wang, *Appl. Catal. B: Environ. Energy* **2025**, *371*, 125248.
[S45] Y. Yang, Y. Sun, Y. Wang, X. Zhang, W. Zhang, Z. F. Huang, L. Yin, A. Han, G. Liu, *J. Am. Chem. Soc.* **2025**, *147*, 8893–8905.
[S46] Z. Wang, X. Lian, R. Yang, X. Guo, S. Wei, J. Zhang, X.-H. Bu, *ACS Catal.* **2025**, *15*, 8230–8238.
[S47] S. M. Varghese, A. V. Gopinathan, S. Rathnakumaran, S. Sasikumar, S. Kunnikuruvan, R. R. Gangavarapu, *ACS Catal.* **2025**, *15*, 5982–5992.
[S48] Y. Li, P. Qiu, C. Qi, M. Li, M. Xie, G. Zhu, W. Luo, *Trans. Mater. Res.* **2025**, *1*, 100030.
[S49] W. Qiu, Y. Guo, X. Z. Fu, J. L. Luo, *Adv. Funct. Mater.* **2025**, *35*, 2415970.
[S50] L. Huang, L. Ye, H. Wang, T. Wei, T. Xia, Y. Du, X. Zhang, Y. Ge, *ACS Appl. Energy Mater.* **2025**, *8*, 5135–5143.
[S51] W. Ye, Y. Yao, X. Wei, M. Xu, S. Zhao, W. Wang, G. Jia, F. Dai, P. Gao, X. Lu, X. Li, B. Xi, N. Wang, S. Xiong, *Angew. Chem. Int. Ed.* **2025**, e202509303.

Table S2. Physical parameters of Fe-HBD, CD@Fe-500, CD@Fe-700, and CD@Fe-900.

Materials	SA_{BET}[a] (m^2/g)	V_{mic}[b] (cm^3/g)	V_{meso}[b] (cm^3/g)	V_t[c] (cm^3/g)
Fe-HBD	1095	0.36	0.042	0.41
CD@Fe-500	282	0.040	0.18	0.22
CD@Fe-700	424	0.048	0.23	0.28
CD@Fe-900	400	0.039	0.23	0.27

[a] Surface area obtained by a BET plot. [b] Micropore and mesopore volume obtained by a t-plot. [c] Total pore volume.

Table S3. The relative energies (in eV) for the intermediates on Fe_3O_4 (311) and α-Fe (110) surfaces in the energy profile.

Intermediates	Fe_3O_4 (311) Relative Energies (eV)	Intermediates	α-Fe (110) Relative Energies (eV)
NO_3^-(*aq*)	0.00000000	NO_3^-(*aq*)	0.00000000
*NO_3	0.09104997	*NO + *O + *O	-5.36852180
*NO_2	-1.50989341	*NO + *O	-4.70682494
*NO	-3.15757527	*NO	-4.17806543
*HNO	-2.67669987	*NH + *O	-7.49954852
*HNOH	-3.33409957	*NH	-6.57803213
*NH	-3.53108919	*NH_2	-6.31540288
*NH_2	-5.52193070	*NH_3	-6.50708613
*NH_3	-7.20167483		
